# Claudin and Rab proteins are key molecular components involved in coccidiosis resistance in Portuguese Merino sheep

**DOI:** 10.1186/s12711-025-01020-x

**Published:** 2025-12-17

**Authors:** Endika Varela-Martínez, Ana Afonso, Dimitra Mainou, Fábio Teixeira, Telmo Nunes, Pedro Vieira, Inês Sarraguça, Cristina Martins, Natalia Campbell, Rafael Cordeiro da Silva, Tiago Perloiro, Luís Madeira de Carvalho, Ana Cristina Ferreira, Luís Telo da Gama, Helga Waap, Andreia J. Amaral

**Affiliations:** 1 Department of Genetics, Physical Anthropology and Animal Physiology, Faculty of Science and Technology, University of the Basque Country (UPV/EHU), Bº Sarriena, 48940//5 Leioa, Spain; 2 Instituto Nacional de Investigação Agrária E Veterinária (INIAV), Quinta Do Marquês, Av. da República, 2780-157 Oeiras, Portugal; 3 Global Health and Tropical Medicine, GHTM, Associate Laboratory in Translation and Innovation Towards Global Health, LA-REAL, Instituto de Higiene E Medicina Tropical, IHMT, Universidade NOVA de Lisboa, UNL, Rua da Junqueira 100, 1349-008 Lisbon, Portugal; 4 Instituto Politécnico de Santarém, Escola Superior de Saúde, Complexo Andaluz, Apartado 279, 2001-904 Santarém, Portugal; 5 Faculty of Veterinary Medicine, Aristotle University of Thessaloniki, Thessaloniki, Greece; 6 MED—Mediterranean Institute for Agriculture, Environment and Development and CHANGE—Global Change and Sustainability Institute, University of Évora, Polo da Mitra, Ap. 94, 7006-554 Évora, Portugal; 7 School of Science and Technology, University of Evora, Evora, Portugal; 8 Faculty of Veterinary Medicine, University José Eduardo Dos Santos, Huambo, Angola; 9 Faculty of Veterinary Medicine, CIISA—Centre for Interdisciplinary Research in Animal Health, University of Lisbon, Avenida da Universidade Técnica, 1300-477 Lisbon, Portugal; 10 Associate Laboratory for Animal and Veterinary Sciences (AL4AnimalS), 1300-477 Lisbon, Portugal; 11 Faculdade de Medicina Veterinária, Universidade Lusófona de Humanidades E Tecnologias, Centro Universitário de Lisboa, Campo Grande, 1749-024 Lisbon, Portugal; 12 Associação Nacional de Criadores de Ovinos de Raça Merina (ANCORME), Évora, Portugal; 13 BioISI—Instituto de Biosistemas e Ciências Integrativas, Faculdade de Ciências, Universidade de Lisboa, 1749-016 Lisbon, Portugal

## Abstract

**Background:**

Although coccidial infection is often asymptomatic in sheep, both clinical and subclinical forms of the disease are linked to considerable production losses, mainly in young lambs. Studies aiming to identify genetic markers for use in selection programs towards increasing genetic resistance to coccidiosis are lacking and have yet to be performed in Portuguese Merino sheep. The purpose of this study was to identify genomic regions associated with resistance to coccidiosis by conducting a genome-wide association study (GWAS) in Portuguese Merino sheep.

**Results:**

From an initial population of 1,022 sheep having known phenotypic characteristics, 206 and 202 distinct animals were genotyped using 50 K and 600 K Single Nucleotide Polymorphism (SNP) arrays, respectively. After the 50 K array was imputed using a 600 K array as reference, an association analysis was performed for faecal oocyst counts (FOC). We identified 12 SNPs that were significantly associated with resistance by using a chromosome-wide significance threshold. The significant SNPs were related to *Ccser1, Thsd4, Eci1, Tnfrsf12a, Chrm3 and Slc20a2* genes. We identified 80 candidate genes located in the proximity of the significant SNPs using the defined confidence regions. Two types of gene set enrichment analyses were performed. Enrichment based on the set of candidate genes, identified the terms virus receptor activity and exogenous protein binding to be enriched, both due to two claudins, *CLDN6* and *CLDN9.* Enrichment based on gene interactions, showed enrichment of terms related to transport vesicles, mainly due to the presence of Rab proteins.

**Conclusions:**

Given the role that Rab and Claudins play in host-parasite relationships, these results suggest the existence of reliable markers associated with resistance to coccidiosis. These markers should be explored in future studies to further validate their use in marker assisted selection, with the goal of enhancing sustainability of the breed conservation-management program.

**Supplementary Information:**

The online version contains supplementary material available at 10.1186/s12711-025-01020-x.

## Background

Coccidiosis is a parasitic disease caused by protozoa of the genus *Eimeria* and poses a significant threat to the health and productivity of sheep flocks worldwide. The disease damages epithelial cells that transport nutrients and fluids from the intestines into the host. Infections can result in dysentery or in more severe infections, haemorrhaging of blood and plasma into the gut lumen, or bacterial infection. Clinical signs of coccidiosis are observed mainly in 4–8-week-old lambs housed with their dams, and 2 to 3 weeks after weaning. Moreover, following a change in diet, especially after entering feedlots, sheep of all ages can develop coccidiosis [1]. Besides clinical coccidiosis, subclinical infection can have a significant negative impact on sheep production, causing reduced growth rate, lowered feed efficiency, increased susceptibility to other diseases, reduced production and, although less common, increased mortality [2]. Costs for control of coccidiosis in sheep have been estimated to be $100 million per year in the United States alone [3].

Current control of coccidiosis in sheep often relies on anticoccidial drugs, with associated costs. Moreover, emergence of drug-resistant strains has reduced treatment efficacy and capacity to control outbreaks. No new drugs in this category have been approved for decades [4]. Overall, drug-resistance problems, as well as consumer’s preference for natural meat products, have driven the search for more sustainable parasite control strategies. Thus, a series of complementary measures that have already put in place to reduce anti-helminthic resistance in ruminants, such as rotational grazing, use of natural anti-parasitic compounds, flock management practices, or dietary supplementation have been proposed and may be useful in integrated coccidiosis control programs [5]. Along with these options, genetic resistance to gastrointestinal (GI) nematodes has been of increasing interest as an alternative parasite control approach in ruminants. Breeding sheep for genetic resistance to coccidia infection is a promising tool to manage coccidiosis in sheep, thus achieving higher sustainability of the production system. Furthermore, selective breeding may bring ethical benefits by reducing the number of animals exposed to disease [6].

Genetic selection of more resistant individuals relies on the existence of genetic variation [7], and previous studies show there is evidence of genetic variation among sheep in resistance to several species of GI parasites, including *Eimeria* [8, 9]. Previous studies of commercial sheep have considered selection of sheep for enhanced resistance to GI parasites [10–12]. Such selection is considered feasible when sheep face natural parasite challenges [13] and breeding for resistance should be considered for introduction into broader control programs [14]. As for other parasite resistance traits, for coccidiosis, FOC is a trait with low heritability, estimated at 0.09 (± 0.03 SE) in Scottish Blackface sheep [8].

Previous studies have proposed that genomic selection is particularly useful for polygenic, lowly heritable traits that have a complex genetic architecture [6, 15]. With the recent dramatic decrease in sequencing and genotyping costs, GS is more readily accessible than ever and has enabled reliable assessment of genetic disease risks and prediction of genomic breeding values [16]. GS can be performed using either all SNPs or only a subset of SNPs obtained from whole-genome sequencing (WGS) or a SNP array. In theory, the accuracy of GS should be higher when using all SNPs from WGS, but in practice this increase is rarely observed [17], and small increases or even a decrease in accuracy have been reported [18, 19]. SNPs that are not informative generate a burden and multiple studies have reported higher GS accuracy when using subsets of SNPs that are associated with the phenotype [20].

A GWAS allows identification of SNPs that are associated with a phenotype. Moreover, identifying candidate genes and corresponding transcription factors (TFs) associated with significant SNPs provides insights into the complex genetic architecture of traits by highlighting relevant biological processes. Previous studies have shown that TFs of genes can be associated with important traits in several livestock species, including sheep [21].

GWAS for identification of SNPs that are significantly associated with GI parasites requires availability of records at the individual sheep level, which are laborious and costly to obtain. Many studies have focused on identification of SNPs associated with faecal nematode egg counts [22–26] but the number of such SNPs identified are few. GWAS studies in chickens with induced coccidiosis identified SNPs located in genomic regions involved in tissue proliferation and repair and in primary innate immune response [27, 28]. GWAS studies focused on FOC are scarce in sheep.

We here present the first GWAS for FOC in Portuguese Merino using genotypes for ~ 550 K SNPs and phenotype data for ~ 400 Portuguese Merino lambs. Portuguese Merino sheep are mostly bred in Southern Portugal and represent an important economic resource in this region. The Merino group includes two breeds known as Black Merino and White Merino, which differ in coat colour and have been raised independently for several decades. Merino sheep are pastured all year round and constantly exposed to a wide range of GI parasites. We hypothesised that a GWAS study in naturally infected Portuguese Merino lambs in this region would allow identification of SNPs significantly associated with GI parasites and with associated biological processes.

## Materials and methods

### Study design

The study was developed as part of project PTDC/CVT-CVT/28798/2017, whose primary goals were to study the epidemiology of GI parasites in Merino sheep and identify SNPs significantly associated with faecal egg counts (FEC). Sample collection was conducted in farms distributed in the regions of *Ribatejo* and *Alentejo* in southern Portugal. A total of 1,022 Merino lambs (354 Merino Black and 668 Merino White) were screened for parasites in the study, distributed across 35 farms, with on average 29.2 ± 9.68 animals sampled per farm. Blood samples were taken by the Merino Producer’s Association (ANCORME) as part of the regular parentage testing program for pedigree confirmation and animal registration. Blood was aspirated into S-Monovette® DNA Exact tubes and a subsample transferred to FTA cards. Faecal samples were collected directly from the rectal ampulla of each animal into plastic bags, individually identified, and stored refrigerated until laboratory analysis. Samples were collected from September 2019 to December 2021. The mean lamb age at sample collection ranged from 171.9 (± 13.8 SD) to 340.8 days (± 34.0 SD). Depending on age and farm, several animals were dewormed between 54 and 261 days before sample collection with antihelmintics commonly used in sheep (benzimidazoles and macrocyclic lactones). See Additional file 1, Table S1, for a more detailed summary per farm.

### Phenotypic measurements

Faecal egg and oocyst counts were performed by the Mini-FLOTAC method [29, 30]. Five grams of each faecal sample were homogenised in 45 ml saturated NaCl solution in a glass beaker and the homogenate passed through a tea strainer into a 50 ml conical tube. For oocyst counting, 1 ml of the filtered homogenate was filled into one of the chambers of the Mini-FLOTAC device, resulting in a sensitivity of 10 oocysts per gram of faeces. All oocysts under the grid of the chamber were counted and multiplied by 10 to obtain the number of oocysts per gram. Levels of FOC infection were classified according to Yan et al. (2021) [26].

### Genotyping and quality control

Since the project prioritised finding SNPs significantly associated with GI nematodes, our initial approach was to select 408 animals with extreme values of faecal egg count (75th and 25th percentile), considering all data and per farm distributions. Coccidia burdens in these animals were assumed to reflect a natural distribution of infection in sheep and no association between FEC and FOC was identified in this subsample of animals selected for genotyping (see Additional file 2: Fig. S1). DNA was extracted using the sbeadex Livestock DNA Purification Kit. (LGC). The GeneSeek® Genomic Profiler™ Ovine 50 K Beadchip (Neogen, Lansing, MI, USA) was used to genotype 206 samples and the remaining 202 animals were genotyped with the Ovine Infinium HD 600 K Beadchip (Illumina Inc., San Diego, CA, USA). The distribution of lambs by breed group and genotyping panel is presented in Table 1. The farms from the genotyped animals were composed of either white or black merino. Thus, breed and farm were not independent variables. The sampling of animals per farm was quite uneven, with the number of animals genotyped per farm averaging 10, ranged from 1 to 25, and included 15 farms with less than 10 animals.

**Table 1:** Distribution of animals genotyped using the 50 K and 600 K SNP Chips

	Merino White Males	Merino Black Males	Merino White Females	Merino Black Females
50 K Chip	11	3	136	56
600 k Chip	19	7	118	58

Quality control of data on both genotype datasets was performed using PLINK v1.9 [32]. Animals with a call rate below 90% were removed. In addition, markers located in sex chromosomes, duplicated in the chip, or with one or more multi-character allele codes were filtered out. Furthermore, SNPs were removed if the missing call rates exceeded 10%, the minor allele frequency (MAF) was less than 1%, or the Hardy–Weinberg equilibrium *p*-value was less than 1E-6. SNP positions were based in the Oar_v3.1 of the sheep genome assembly [33]. Prior to imputation, the conform-gt program (included in Beagle) was used to verify that the markers shared between the target and reference VCF files had identical genomic positions and allele designations. Then, the 50 K array was imputed using Beagle v5.4 [34], setting the quality approved genotypes obtained, using the 600 K array as reference. The imputed 50 K and 600 K datasets were combined and a second step for genotype quality validation and filtering was performed using the same criteria as described above. Next, SNPs in linkage disequilibrium (LD) were discarded from the dataset using PLINK, using a window of 50 SNPs, a window shift of five SNPs, and a *r*^*2*^ greater than 0.5. The dataset obtained at this stage was used in the following analyses.

### Genome-wide association analysis

Prior to the GWAS, a principal component analysis (PCA) was performed to check how the imputed dataset projected into the PCA space computed for the reference panel, and to check patterns and stratification of the data. The PCA was performed on the reference 600 K array and the imputed 50 K array was projected into it.

Association analysis for FOC was performed using the genome-wide complex trait analysis (GCTA v1.94.1) software tool [35]. Association analysis for FEC is ongoing and will not be presented here. The mixed linear model used for FOC was:

1 $${\mathbf{y}} = {\mathbf{\mu }} + {\mathbf{X}}_{1} \varvec{\beta} _{1} + {\mathbf{X}}_{2} \varvec{\beta} _{2} + {\mathbf{X}}_{3} \varvec{\beta} _{3} + {\mathbf{X}}_{4} \varvec{\beta} _{4} + {\mathbf{g}} + ~{\mathbf{f}} + {\mathbf{e}}$$

where **y** is a vector of FOC values, **µ** is the mean term, **β**_**1**_**, β**_**2**_, and **β**_**3**_ are vectors of fixed effects, which included the linear effect of age (in days), sex, and season at sample collection, and **β**_**4**_ is the fixed additive effect of the SNP to be tested. **X**_**1**_, **X**_**2**_, **X**_**3**_, and **X**_**4**_ are the corresponding design matrices linking observations in **y** to these fixed effects. **g** is a vector of the polygenic genetic effects of the individuals, **f** is the random effect of farm, and **e** is a vector of residual effects. The residuals and total genetic effects were assumed to follow a normal distribution N(0, **I**σ_e_^2^) and N(0, **G**σ_u_^2^), respectively, where **G** is interpreted as the genomic relationship matrix between individuals [35].

Significance thresholds for GWAS have been usually calculated using a *Bonferroni* multiple test-correction, but such adjustments can be conservative due to influence of LD between genetic markers [36]. Thus, a chromosome-wise significance threshold was calculated by the method named “simple M”, which was proposed by Gao et al. (2008) [32] and has been shown to be an efficient alternative when more computationally intensive approaches such as permutation are not feasible [38]. Briefly, the effective number of independent markers for each chromosome (M_eff_C_) was calculated as the number of markers needed so that the eigenvalues derived from the composite LD correlation matrix explain 99.5% of the variation. Then, the *Bonferroni* correction formula was applied to calculate chromosome-wise significance as α_C_ = 0.05/M_eff_C_. The total number of independent markers in this study was 229,235. The set of markers with a *p*-value lower than chromosome-wise significance were used for further analyses.

The genomic inflation factor (λ) estimates the amount of inflation by comparison of observed test statistics to those expected under the hypothesis of no effect [39]. Chi-square test statistics were calculated from *p*-values assuming one degree of freedom and λ was calculated as the median of observed chi-squared test statistics divided by the expected median [40]. A value of the genomic inflation factor close to unity reflects no evidence of inflation.

### Post GWAS analyses

Haplotype blocks were estimated using PLINK and those with significant SNPs from the GWAS analyses were used for further analyses. Coordinates of significant SNPs were updated from the Oar_v3.1 assembly to the ARS-UI_Ramb_v2.0 assembly [41] using SNP variant information from Ensembl. Haplotype blocks were updated using the UCSC LiftOver tool [42]. Those SNPs that were unable to be re-mapped or haplotype blocks whose coordinates were only partially transferred (e.g., fragmented or spanning multiple regions in the new assembly), were excluded to avoid inconsistencies in SNP composition or inter-marker distances. The Genomic Annotation in Livestock for positional candidate loci (GALLO v1.5) R package was used for annotation of genes and Quantitative Trait Loci (QTLs) that were in proximity to candidate markers. The Animal QTLdb database (release 53) for sheep was used for the analysis [43]. Candidate genes were considered if their boundaries were within 100 kbp upstream or downstream of significant SNPs. The LD decay distance, defined as the physical distance at which the average LD (r^2^) between markers drops to approximately 0.2, has been reported to be similar among different sheep breeds—approximately 100 kbp—by Marina et al. (2021) [44]. This value was therefore used to define regions of confidence in the present study. In addition, the AnimalTFDB 4.0 database was used to check whether any TFs or co-transcription factors were associated with the genes located within the confidence regions [45]. Enrichment analyses for gene ontology (GO) terms were performed with the gprofiler2 R package [46] forthe candidate gene list composed of genes within the confidence regions defined above, using the False Discovery Rate (FDR) multiple testing correction method with an FDR cut-off value of 0.05. Remaining parameters were left as default. Furthermore, the GeneMANIA (v3.5.3) Cytoscape plugin for the[47] was used to identify the most related genes to the set of genes located in the confidence regions. For that purpose, orthologous human genes were searched with biomaRt [48] and the resulting list of orthologous genes were passed to GeneMANIA and run with default parameters, which uses the query gene-based method as weights, in which interactions among the query gene list are prioritized over genes outside the list. Genes without an orthologous sheep gene were removed from the network.

Overrepresentation of transcription factor binding sites (TFBSs) in the regulatory regions of genes located within confidence regions was performed using the Transcription Factor Matrix Explorer (TFM-explorer v2.0) program [49]. Since transcription start sites (TSSs) are uncertain for some genes in the current assembly, the sequences from 3,000 bp upstream to 300 bp downstream of genes’TSSs were extracted [50], based on the ARS-UI_Ramb_v2.0 assembly. These sequences were used as input for TFM-explorer, which was executed using all vertebrate weight matrices from the JASPAR database [51]. An enrichment analysis for GO terms was performed with gprofiler2 [46].

## Results

### Genotype quality control and imputation

After quality control of the genotypes of the 50K chip samples, 11 samples were removed due to a low call rate. From the initial set of 48,841 autosomal and bi-allelic SNPs, missing call rates greater than 10% were found for 1050 SNPs, a MAF lower than 1% was detected for 2,486 SNPs, and a Hardy–Weinberg equilibrium *p*-value less than 1E-6 was found for 340 SNPs. Thus, genotypes for 44,965 SNPs for 195 sheep remained for further analyses.

Following the same quality control criteria for genotypes from the 600K chip samples, one sample was removed due to low call rate. From the initial set of 577,205 autosomal and bi-allelic SNPs, 12,162 SNPs were removed due to missing call rate, 25,033 SNPs were filtered out due to MAF thresholds, and 2,291 SNPs were removed due to Hardy–Weinberg equilibrium. Finally, genotypes for 537,719 SNPs for 201 sheep remained for further analyses.

The 50K chip dataset was imputed using the 600K chip as reference. From the 44,965 SNPs in the 50K chip data, only 34,992 SNPs had corresponding markers in the 600K chip. After imputation was performed and both VCF files were combined, quality control was performed with the previous filtering criteria, resulting in 2,709 and 3,839 SNPs filtered-out due to Hardy–Weinberg equilibrium and MAF, respectively. The resulting dataset used for GWAS was composed of genotyped for 531,171 SNPs for 396 sheep.

### Phenotype statistics and population structure

FOC is a measure of the intensity of coccidia infection. Coccidia prevalence was high, with 88.1% of the genotyped samples with a value greater than 0. The average FOC value was 923.7 (± 1,768.2 SD), the highest value being 13,570 oocysts/gram of faeces. We had 47 sheep with no infection (FOC = 0 oocysts/gram); 307 with a mild infection (FOC < 1800 oocysts/gram) and an average FOC value of 517.1 (± 434.5 SD); 29 with a moderate infection (FOC = 1800–6000 oocysts/gram) and an average value of 3,155.9 (± 1,420.7 SD); and 13 with a severe infection (FOC > 6000 oocysts/gram) and an average value of 8,886.2 (± 2,256.3 SD). Most samples showed a mild infection.

Prior to the GWAS analysis, a PCA was performed to investigate population stratification (see Additional file 3: Fig. S2). Each principal component explained a small portion of the variance in the reference array, with PC1 explaining 2.5% and PC2 explaining 1.8%. Genotype imputation had no structural effect on the data. Clustering by breed-group was observed.

### SNPs associated with FOC based on GWAS and gene annotation

The selected GWAS model displayed a genomic inflation factor (λ) of 0.924 and included age, sex, and season as fixed effects and farm as random effect (see Additional file 4: Fig. S3). Other models were analysed such as removing age, sex, or season as fixed effects, which generated genomic inflation factors slightly lower than the selected model (see Additional file 5: Fig. S4). Since breed is correlated with farm, we evaluated whether including breed as a fixed effect in addition to age, sex, season (fixed), and farm (random) would improve the model fit (see Additional file 5: Fig. S4e). The *p*-values from the selected model and the other investigated models were highly correlated (see Additional file 6: Fig. S5) and inclusion of breed as a fixed effect had negligible impact on SNP associations (Pearson correlation of –log₁₀(p) values between models = 0.995; genomic inflation factor λ = 0.9245 vs. 0.9237; see Additional file 6: Fig. S5e). Given the minimal differences, we retained the simpler model (age, sex, season fixed; farm random) for the final analyses.

**Fig. 1:**
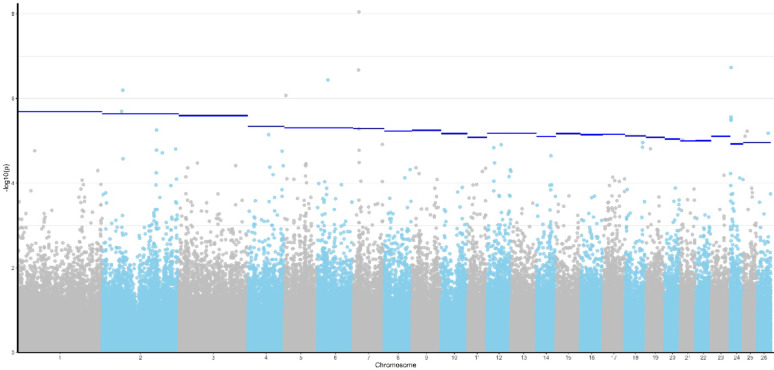
Manhattan plot showing genome-wide association results for fecal oocyst count (FOC) in sheep naturally infected with *Eimeria* (coccidia). Blue line represents the chromosome-wide significance thresholds

GWAS allowed identification of 14 significant SNPs at a chromosome-wide significance level. Since two of those SNPs could not be re-mapped to the ARS-UI_Ramb_v2.0 assembly, we ended up with 12 significant markers (Table 2, Fig. 1). The chromosome-wide significant thresholds employed are shown in Additional file 7, Table S2. Box plots showing the effect estimates of significant SNPs are in Additional file 8, Fig. S6. Most significant SNPs were located within introns of known genes (namely: *Ccser1, Thsd4, Eci1, Tnfrsf12a, Chrm3 and Slc20a2*), one had a missense predicted consequence on a transcript from *Znf200,* and a few SNPs located downstream of non-coding genes. Two significant SNPs were located within an intergenic region.

**Table 2:** Significant SNPs for Coccidia FOC

SNP rsID	Chr	bp	A1	A2	Freq	p-value	Gene	Consequence type
rs411343808	2	63,607,030	G	A	0,0202	2,00E-06	ENSOARG00020032169	downstream
rs417924701	5	4,799,630	A	C	0,0354	8,44E-07		intergenic
rs422982057	6	34,899,299	G	A	0,0101	3,63E-07	CCSER1	intron
rs411549257	7	17,031,457	G	A	0.0467	2,12E-07	ENSOARG00020027093	downstream
rs417935219	7	18,191,375	A	G	0.0341	8,99E-09	THSD4	intron
rs414033502	21	8,293,837	A	C	0.0265	9,63E-06	ENSOARG00020036475	intron
rs415434348	24	2,294,317	G	A	0.0442	2,72E-06	ECI1	intron
rs425217825	24	2,937,586	A	G	0.0139	3,08E-06	ENSOARG00020026408*^1^	synonymous/intron
rs161410499	24	3,214,651	G	A	0.0366	3,26E-06	ZNF200	missense
rs413352396	25	5,145,396	A	G	0.0758	7,78E-06		intergenic
rs418654751	25	11,926,932	A	G	0.0177	5,92E-06	CHRM3	intron
rs418055519	26	36,653,829	A	G	0,0290	6,61E-06	SLC20A2	intron

Freq = frequency of allele A1; Gene = gene with a predicted consequence of the SNP in Ensembl; Consequence type = predicted consequence in Ensembl

^*^^1^Orthologue to cow TNFRSF12A

We identified additional genes within the confidence region of each SNP (see methods). This strategy enabled identification of TFs for the gene list of *Jund, E4f1*, *Znf213*, *Znf200,* and *Znf263,* and co-transcription factors for *Pik3r2* and *Tle3*. Together with genes that overlapped with the significant SNPs, the list of candidate genes included 80 genes (see Additional file 9: Table S3).

### Gene set enrichment analyses and Gene-TF networks

**Fig. 2:**
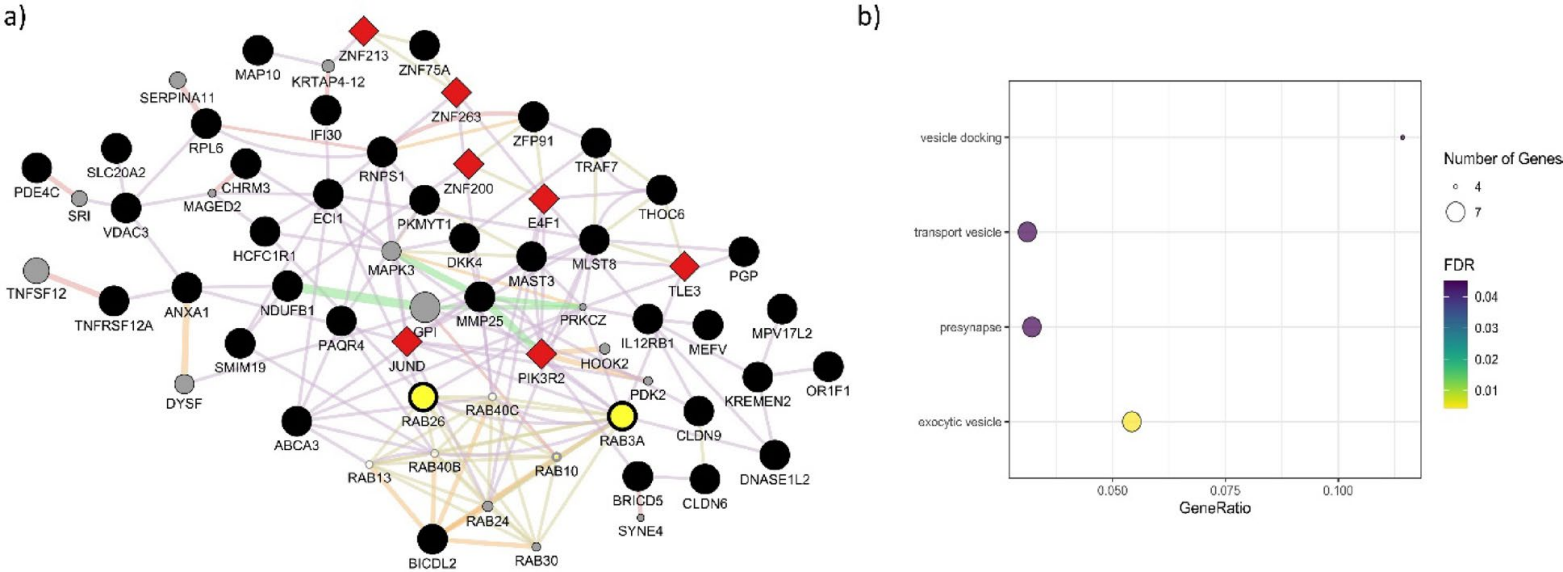
Gene–gene interaction network and enrichment of cellular components and biological processes. (**a**) Gene–gene interaction network. For visualization purposes, nodes without connections to other nodes were removed. Edge colour represents type of relationship between nodes: purple, co-expression; green, genetic interactions; orange, predicted; grey, shared protein domain; and red, physical interaction. Node size represents the degree (number of connections) of the node. Red diamond shaped nodes are genes classified as transcription and co-transcription factors in AnimalTFDB 4.0 database. Yellow nodes are genes from the transport vesicle GO term. Black nodes are genes located in confidence regions of significant SNPs, while grey nodes are genes that are predicted to be functionally related to the query genes. (**b**) Enrichment analysis based on the gene–gene interaction network. The bubble plot shows the enriched terms in the Y-axis and the gene ratio (gene ratio = number of genes from network/all genes included in the term). Bubble size and colour represents number of genes and significance (FDR) in the GO term, respectively

Our analyses only identified one previously defined QTL related to ear size [52], which overlapped the confidence region around the rs161410499 marker. The obtained list of 80 candidate genes was further used to investigate enrichment of molecular functions and gene-TF networks. Two types of enrichment analysis were performed. The first analysis involved testing for enrichment in a gene list and allowed identification of two molecular functions, virus receptor activity (GO:0001618, FDR = 0.0026) and exogenous protein binding (GO:0140272, FDR = 0.0026). Both terms were enriched due to the presence of two claudin family members in the gene list, *CLDN6* and *CLDN9*, as well as a solute carrier family member, *SLC20A2*. The second analysis considers interactions between genes within the query list and other possible gene interactions available in the databases s (Fig. 2a). This analysis identified the presynapse (GO:0098793, FDR = 0.045) cellular component GO term and vesicle related GO terms such as vesicle docking (GO:0048278, FDR = 0.045) in the biological process category, and exocytic vesicle (GO:0070382, FDR = 0.044) in the cellular component category (Fig. 2b). Figure 2a shows that 45 of the identified candidate genes were found to interact (co-expression, genetic relationship or sharing protein domains) and formed a single network cluster. The network contains six of seven genes associated with vesicle related functions, as shown in Fig. 2a. These genes are from the same gene family, *RAB*, which belong to the RAS superfamily of small GTPases [53]. The genes that displayed higher levels of interactions were *RAB3A* and *RAB26*. The network shown in Fig. 2a also shows interactions between genes and TFs, which included several zinc finger proteins, including ZNF200 (with a tolerated missense SNP) and co-transcription factor *PIK3L*.

**Fig. 3:**
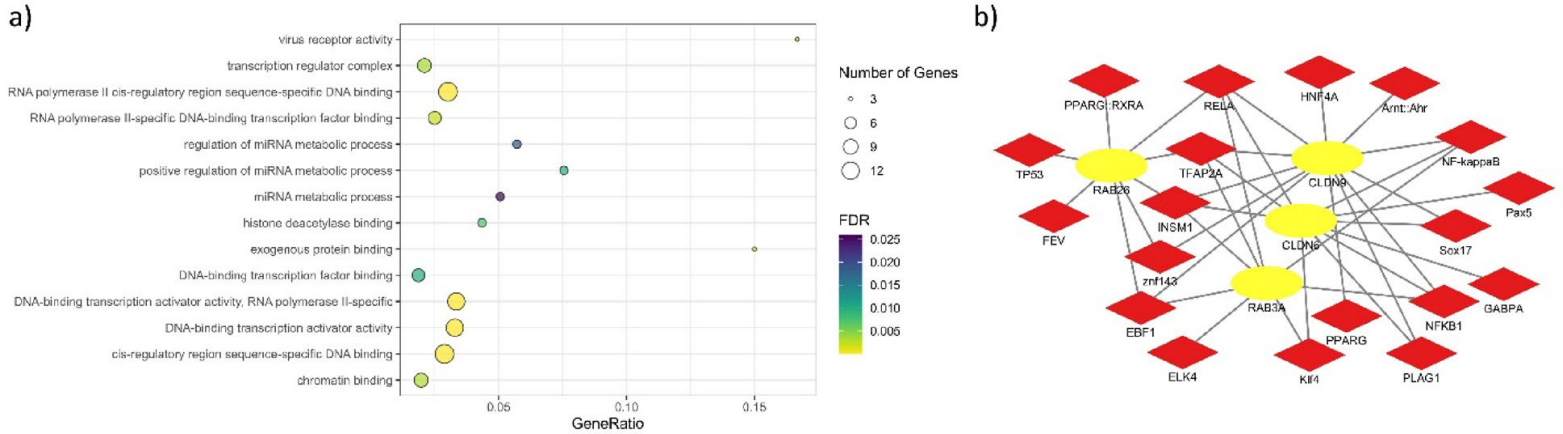
Enrichment of transcription factor binding sites (TFBS) and predicted TF–gene interaction network for candidate genes within confidence regions. (**a**) Enrichment results with genes located in the confidence regions and transcription factors with overrepresentation of TFBSs in their regulatory regions. (**b**) Network representing the enriched genes in the serine-type endopeptidase inhibitor activity GO term and transcription factors with predicted TFBSs in those genes.

Additionally, overrepresentation of TFBSs in regulatory regions of genes located within the identified confidence regions was assessed. This analysis showed overrepresentation of binding sites for TFs of *TFAP2A*, *NFKB1*, *INSM1*, *NK-κβ*, *RelA*, *EBF1*, *GABPA*, *PLAG1,* and *ELK4*, among others (see Additional file 10: Table S4). The enrichment analysis showed terms associated to DNA-binding transcription factor activity (GO:0003700, FDR = 1.96281E-09) and virus receptor activity (GO:0001618, FDR = 0.000873382) (Fig. 3a). The predicted interactions of *RAB3A*, *RAB26, CLDN6,* and *CLDN9* with TFs were represented as a network, showing interactions with NFKB1, NF-kappaB, and RELA (Fig. 3b).

## Discussion

Portuguese Merino is a sheep breed of high economic value and these animals are typically kept in extensive system. Debilitation of flocks from coccidiosis is prevalent among these sheep and resistance to anticoccidial drugs is a major problem. Alternative anticoccidial approaches are needed. In this regard, our study utilized GWAS to identify genetic markers associated with the biological response of lambs to coccidial infection. Given the sample size, results from the two merino types were combined to increase the sample size and improved capability for GWAS detection of significant SNPs. When black and white merino samples were analysed separately, much fewer significant SNPs were identified, most likely due to the low sample size of each merino type.

In total, GWAS identified 12 SNPs that were significantly associated with FOC in lambs naturally exposed to parasites. Seven of these SNPs are located within the introns of genes, two are intergenic, two are located downstream of non-coding genes, and one has a tolerated missense predicted consequence. By considering the region of confidence of each SNP, our analyses retrieved additional candidate genes, reaching a final list of 80 candidate genes. The enrichment analysis identified enrichment of genes from the claudin family, which are integral components of tight junctions and regulate paracellular permeability in epithelial tissues [54]. Forty-five of the 80 candidate genes have different types of interactions reported in the literature, namely, co-expression, sharing of protein domains, and physical interactions. The enrichment analysis that integrates gene interactions identified enrichment of genes encoding Rab proteins, which are members of the RAS superfamily of small GTPases. Rab proteins recruit specific sets of effector proteins onto membranes in their GTP-bound state and coordinate consecutive stages of transport, such as vesicle formation, vesicle and organelle motility, and tethering of vesicles with target membranes [53, 55].The significance of the role that these members of the claudin and the RAS superfamily of small GTPases might play in sheep susceptibility to coccidiosis is outlined below.

Coccidiosis in sheep is an intestinal infection caused by different species of intracellular protozoans of the genus *Eimeria*. Non-sporulated oocysts are excreted onto grazing areas in the faeces of infected sheep. Under optimal conditions of moisture, oxygen, and temperature (24º to 32ºC), these non-infective stages require a minimum of two to five days to sporulate and become infective. Oocysts are usually unable to survive temperatures below − 30ºC or above 40ºC, but this wide range of temperatures allows for sporulated and non-sporulated oocysts to maintain their viability for more than a year, under proper environmental conditions, posing a constant source of infection to grazing livestock. Sporulated oocysts are ingested by sheep and eight sporozoites are released from each oocyst during passage through the intestine due to the action of bile and trypsin. After invading enterocytes, the parasite undergoes a complex cycle of asexual (merogony) and sexual (gametogony) multiplication, resulting in the production of more oocysts, which are shed into the environment with the faeces. The entire life cycle within the host takes 2 to 3 weeks [56], during which the parasite needs to penetrate a diverse range of host tissue barriers, acquire nutrients, and evade host response. The intestinal barrier is the primary defence against harmful substances and pathogens, whose integrity and impermeability are regulated by tight junctions. Claudins that are in the list of candidate genes, such as *CLDN6* and *CLDN9,* are transmembrane proteins that are involved in maintaining the integrity of epithelial cell layers in the intestinal lining [57]. Claudins are integral components of tight junctions between epithelial cells, contributing to the selective permeability of the intestinal barrier. We hypothesize that changes of expression of *CLDN6* or of *CLDN9* affects the permeability of the intestinal epithelium, allowing for pathogens to promote their translocation and invade the host [58]. In fact, infection with the protozoan parasite *Cryptosporidium parvum* results in a substantial downregulation of *CLDN4* in mouse [59]. Chickens infected with *Eimeria tenella* show upregulated expression of *CLDN2* and decreased expression of *CLDN3* and E-cadherin genes in the intestine [60]. A study on dairy calves infected with *Eimeria bovis* observed changes in the expression of *CLDN4* in cecum [61]. Coccidiosis is also known to trigger inflammatory response in the intestine. Other studies have shown that increased inflammation can affect the expression of claudins, including *CLDN6* and CLDN9 [62].

The gene interaction network identified a hub composed only of RAB proteins, two of which, *RAB3A* and *RAB26,* are in the list of GWAS candidate genes and display the highest level of interactions in the network. Rab proteins are a family of small GTPases that play crucial roles in regulating vesicular trafficking and membrane dynamics within eukaryotic cells [63]. Previous studies have shown that apicomplexan parasites manipulate Rab GTPases to modulate cellular trafficking pathways, in order to move host vesicles near to their parasitophorous vacuole membrane, scavenge nutrients from host vesicles, and control host phagocytic activity [64]. For example, *Toxoplasma gondii* is auxotrophic for many metabolites and the parasite scavenges sphingolipids from the host Golgi by sequestering Golgi-derived RAB GTPases such as *Rab14*, *Rab30,* and *Rab43* into the parasitophorous vacuole [65]. *Plasmodium berghei* hijacks the Golgi structure of the hepatocyte and interaction with *Rab11a* is critical for the ability of the parasite to induce Golgi fragmentation, in order to increase nutrient acquisition surfaces [66]. In addition to their vesicle related functions, Rab GTPases modulate innate immunity by regulating transmembrane signals’ transduction [67]. For example, *Rab14* overexpression protects *Plasmodium spp.* sporozoites from degradation in macrophages, while silencing *Rab14* results in increased phagocytosis [68]. Our results showed that SNPs identified by the GWAS are located within genes involved in genetic interactions, in which *RAB3A* and *RAB26* play a crucial role, given their large number of interactions with other genes of the network, which include TFs such as *PIK3R2*, which functions as part of the PI3K pathway, regulating cell growth, proliferation, and survival. *RAB3A* and *PIK3R2* were found to be co-expressed in human cancers [69]. The involvement of *Rab* proteins, specifically *RAB3A* and *RAB26*, in the context of coccidiosis, has not been widely studied. Given the known roles of these proteins, both RAB3A and RAB26 proteins likely play essential roles in the intracellular trafficking of both the *Eimeria* parasite and host immune cells. Modulation of *RAB3A* expression, with its role in vesicle fusion and neurotransmitter release, is crucial for the parasite, since it allows parasite invasion and immune cell modulation, while modulation of *RAB26* expression by the parasite, with its broader vesicular trafficking role, may assist in parasite replication and immune response during *Eimeria* infection.

Additionally, identification of the gene-transcription factor network in our study showed enrichment for DNA-transcription factor activator activity and RNA polymerase II-specific molecular functions. In fact, within this network, we identified interactions of *NF-κB* with *RAB3A, CLDN6,* and *CLDN9*. *NF-κB* plays a critical role in mediating responses to a notable diversity of external *stimuli*. It is a significant orchestrator of immune response, including specific changes of NF-κB levels resulting from parasite infections [70]. Previous studies have shown enhanced NF-κB activity in mice infected with *Trichuris muris*, followed by increased levels of several cytokines [71]. Modulation of macrophage regulation by NF-κB was also observed during* Trichinella spiralis* and *Brugia malayi* infections [72, 73]. Moreover, the *RelA* gene was also identified as a TF that interacts with *RAB3A and RAB26,* which encode one of five proteins that may dimerize to form NF-κB complexes [74].

## Conclusions

**Fig. 4:**
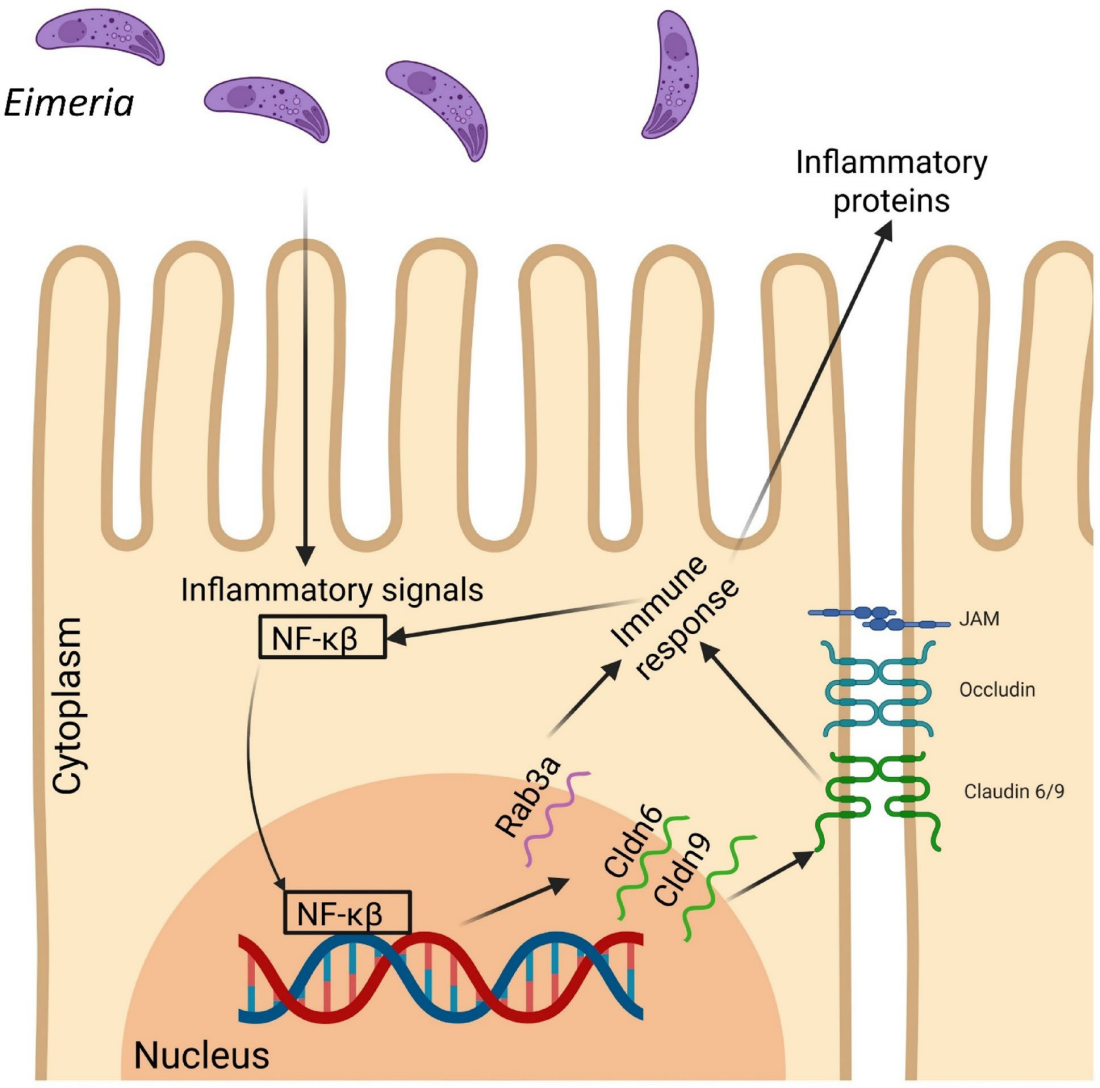
Proposed molecular mechanism underlying resistance to *Eimeria* (coccidia) infection in sheep. Schematic representation of the hypothesized host cellular response to *Eimeria* infection. Activation of the *NF-κB* signaling pathway triggers the expression of immune and inflammatory mediators, including genes such as *RAB3A, CLDN6,* and *CLDN9,* which may strengthen epithelial barrier integrity and coordinate vesicle trafficking involved in immune signalling. Tight junction proteins (Claudins, Occludin, JAM) contribute to maintaining epithelial cohesion and preventing parasite invasion, thereby enhancing resistance to coccidial infection. Created in BioRender. Varela Martínez, E. (2025) https://BioRender.com/1uygi0q

This study has identified GWAS SNPs that maybe in LD with genes that have the potential to establish a mechanism for resistance to coccidia infection in sheep. Our hypothesis (Fig. 4) is that *NF-κB* and *RELA* activate transcription of *CLDN6* and *CLDN9*, which are required to tightening junctions to block parasite entry. Then, *CLDN6/9* can activate *RAB3A*, enhancing junctional vesicle recycling or stabilization, boosting barrier function. *RAB3A* can then can signal to *RAB26*, which activates immune-modulatory responses (possibly recruiting or activating local immune cells). Other TFs in the network (e.g., *PPARG, TFAP2A, ZNF143*) may modulate expression for homeostasis or fine-tune the response depending on infection stage. To date, there have been no programs for breeding of resistance to GI parasites in this breed. Given, the number of SNPs, their statistical significance and functional impact, we hypothesize that Portuguese Merino sheep have undergone natural selection, that increased allele frequencies of genotypes associated with tolerance to FOC. This hypothesis is further supported by the fact that a previous study using a larger GWAS sampling size of Blackface sheep, that did not find any significant association of SNPs with FOC [26].

In conclusion the identified markers may be useful as a basis to further exploit genetic resistance to GI parasites, and not only in Portuguese Merino sheep. We have identified a mechanism that should be further investigated. Validation of the predicted interactions may also enable the identification of novel therapeutic targets. In future studies, it will be important to clarify the role of these SNPs in this physiological mechanism responsible for resistance or susceptibility to coccidiosis in sheep.

## Supplementary Information

Below is the link to the electronic supplementary material.

Supplementary Material 1

Supplementary Material 2

Supplementary Material 3

Supplementary Material 4

Supplementary Material 5

Supplementary Material 6

Supplementary Material 7

Supplementary Material 8

Supplementary Material 9

Supplementary Material 10: Boxplots showing genotype distribution for the significant SNPs identified in this study. The marker ID can be seen at the bottom. The genotype in the x-axis and FOC values in the y-axis. n=number of Merino sheep.

## Data Availability

The dataset has been deposited in the European Variation Archive (EVA) under the study accession number PRJEB79893.
