# Supplementary figures and images for: Claudin and Rab proteins are key molecular components involved in coccidiosis resistance in Portuguese Merino sheep
Source: Genet Sel Evol. 2025 Dec 17;58:2. doi: 10.1186/s12711-025-01020-x (PMC12784492; doi:10.1186/s12711-025-01020-x)

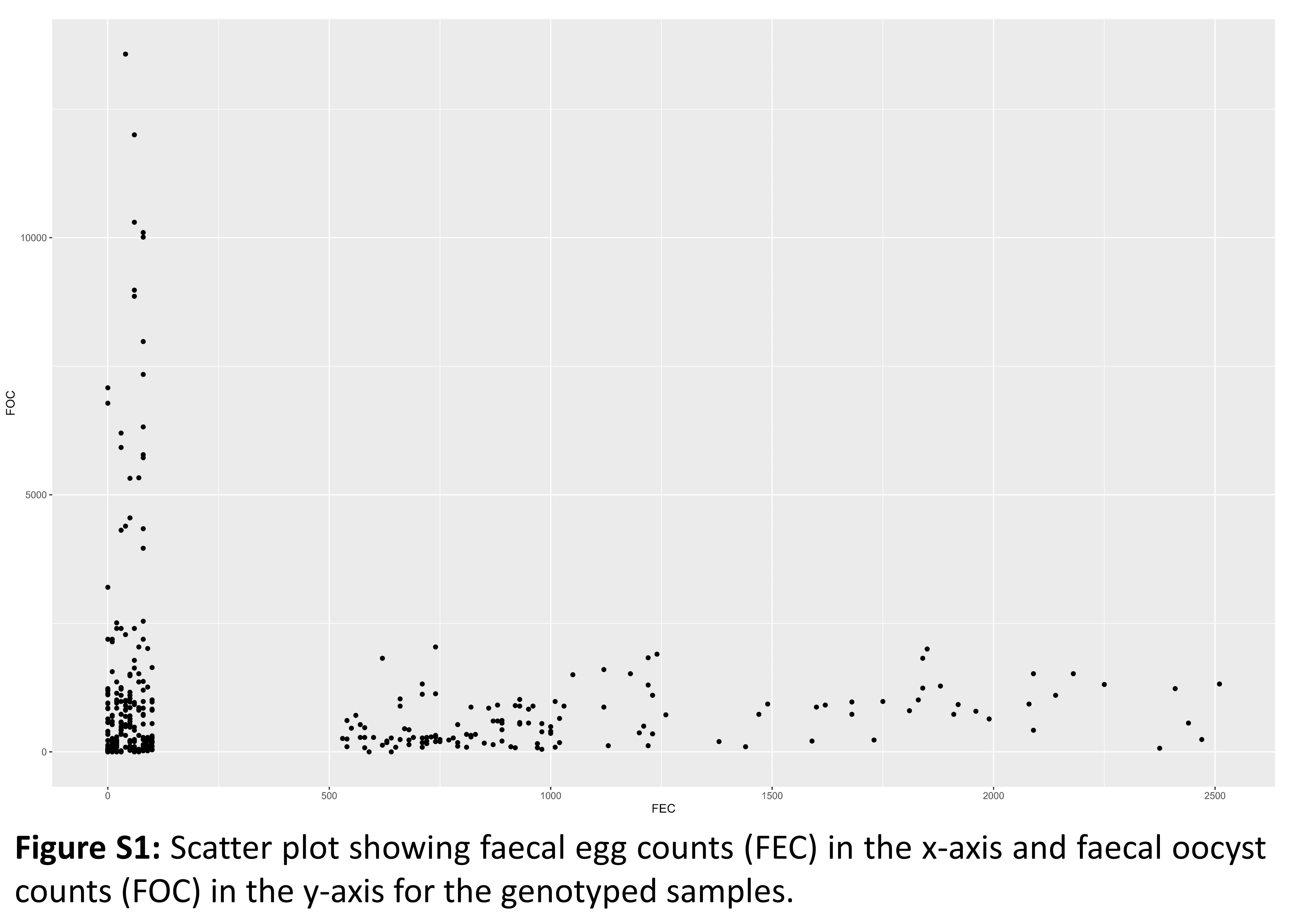

Supplement: Supplementary file 5 — Supplementary Material 5 [file 12711_2025_1020_MOESM5_ESM.jpg]

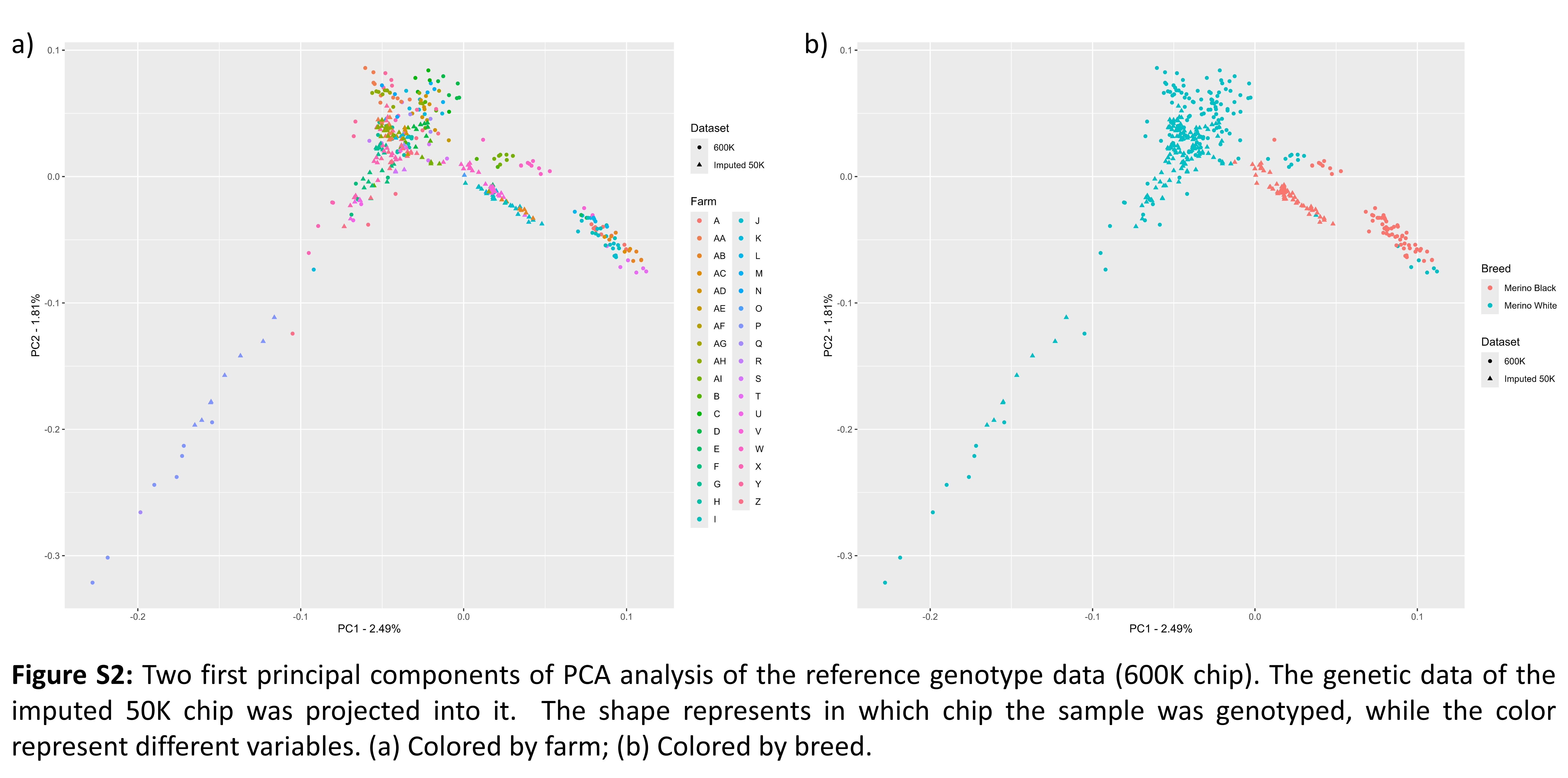

Supplement: Supplementary file 6 — Supplementary Material 6 [file 12711_2025_1020_MOESM6_ESM.jpg]

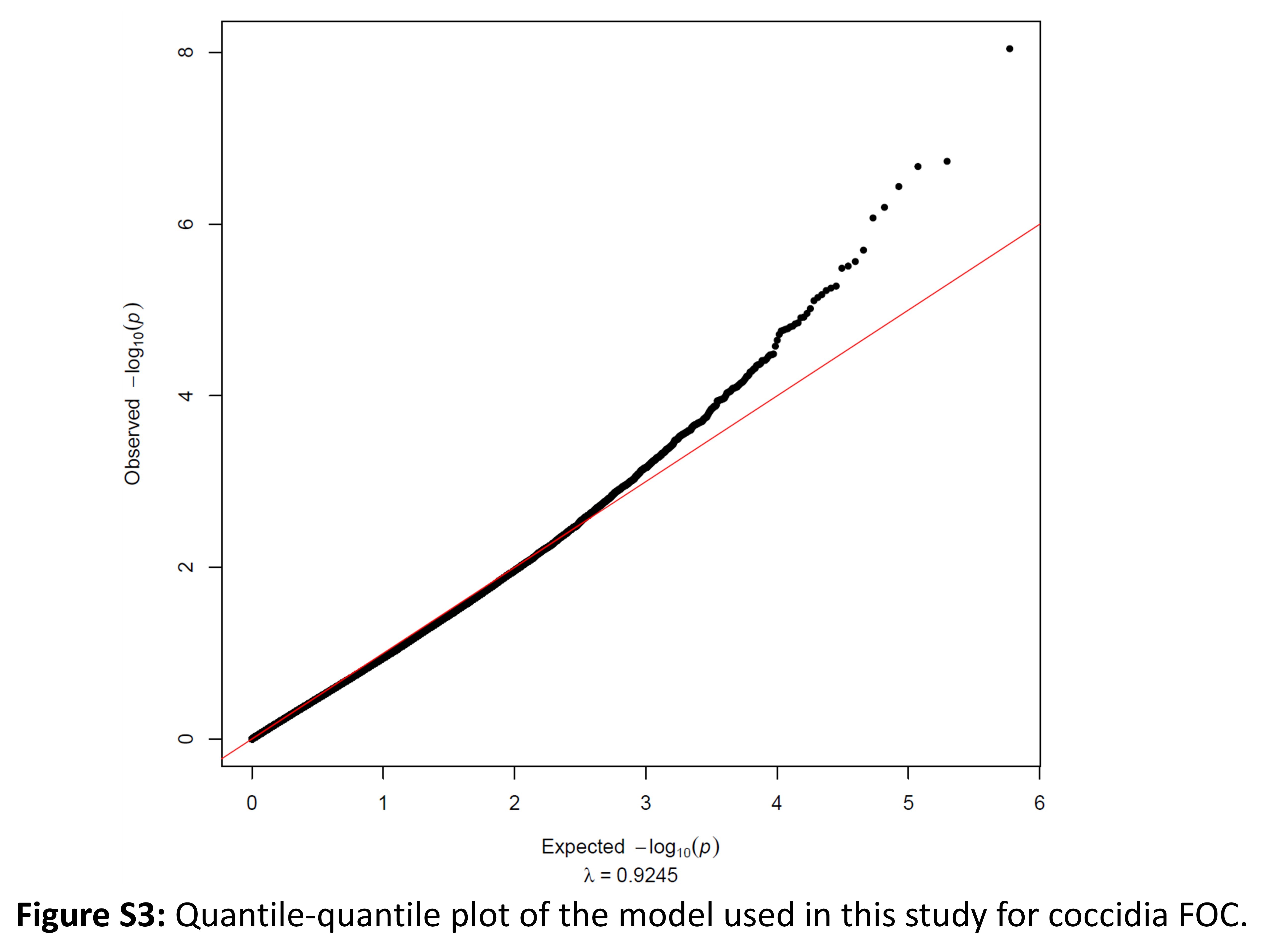

Supplement: Supplementary file 7 — Supplementary Material 7 [file 12711_2025_1020_MOESM7_ESM.jpg]

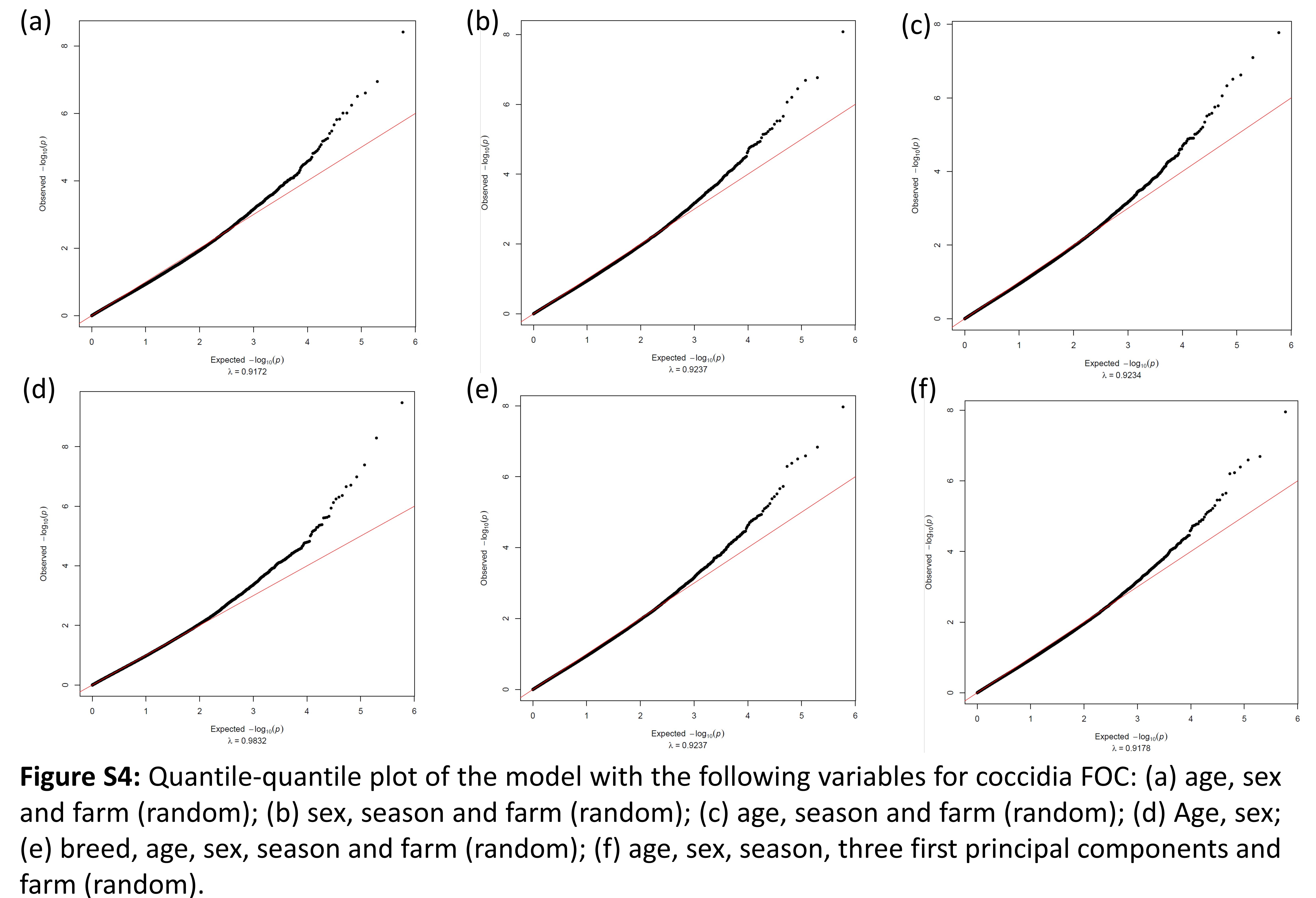

Supplement: Supplementary file 8 — Supplementary Material 8 [file 12711_2025_1020_MOESM8_ESM.jpg]

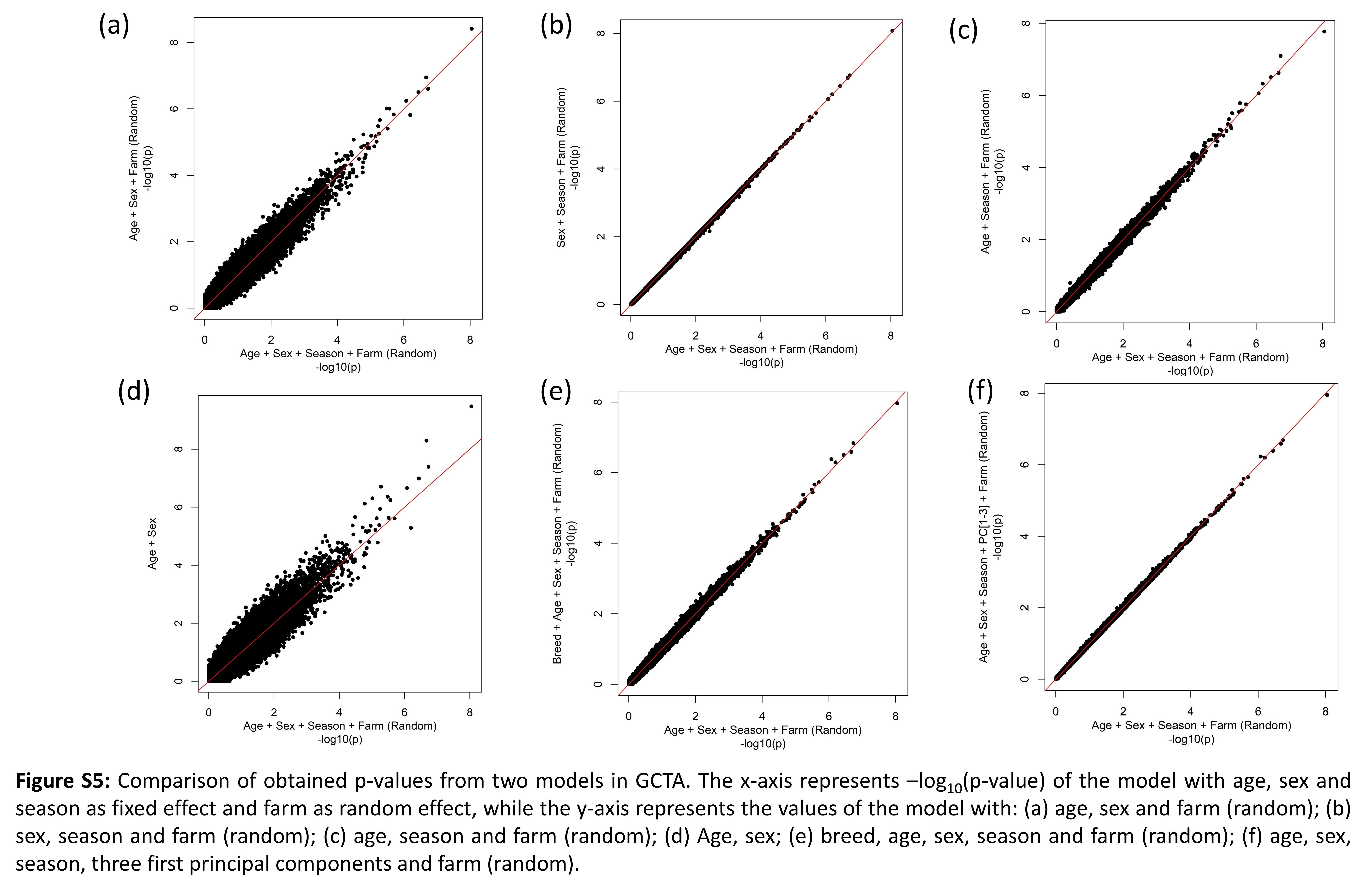

Supplement: Supplementary file 9 — Supplementary Material 9 [file 12711_2025_1020_MOESM9_ESM.jpg]

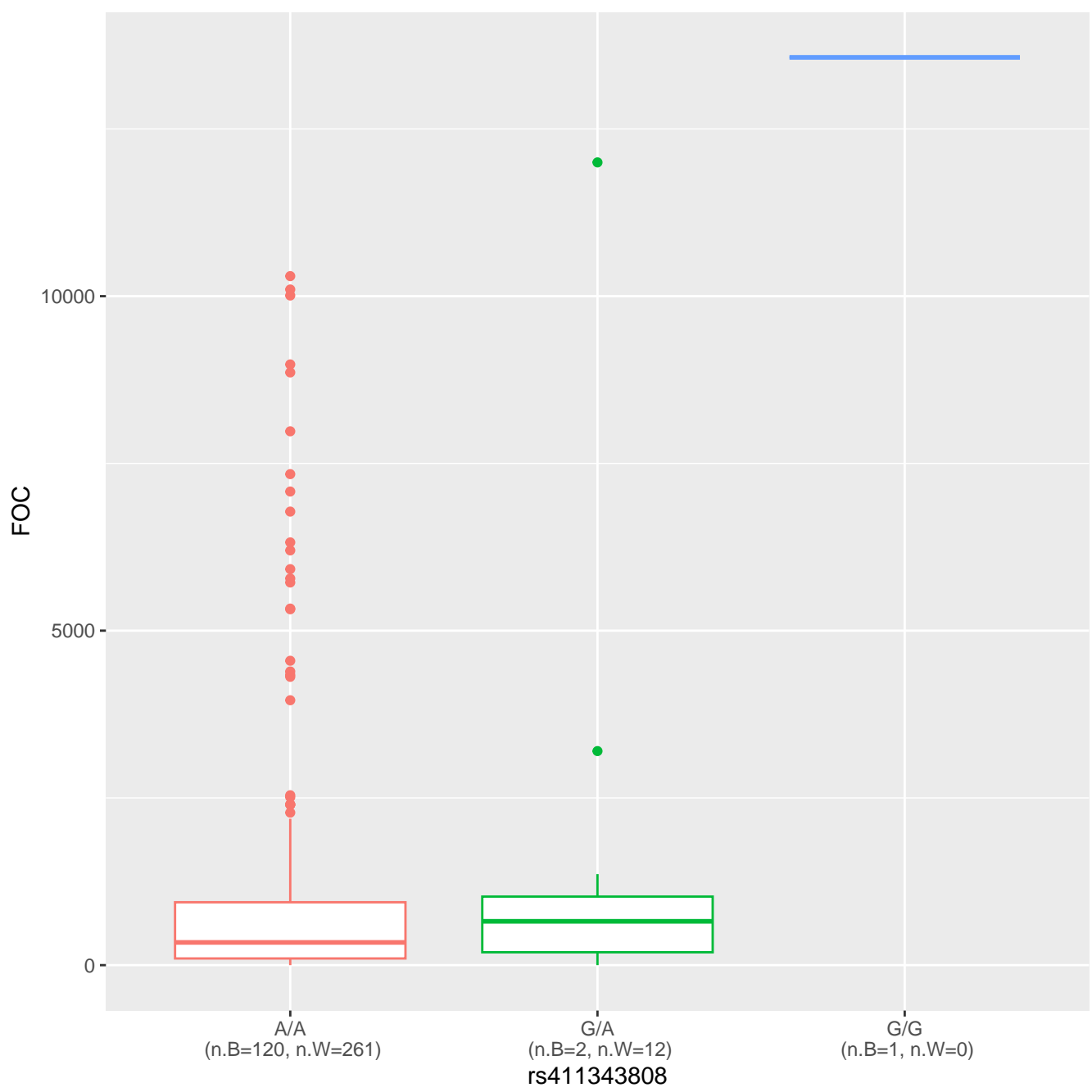

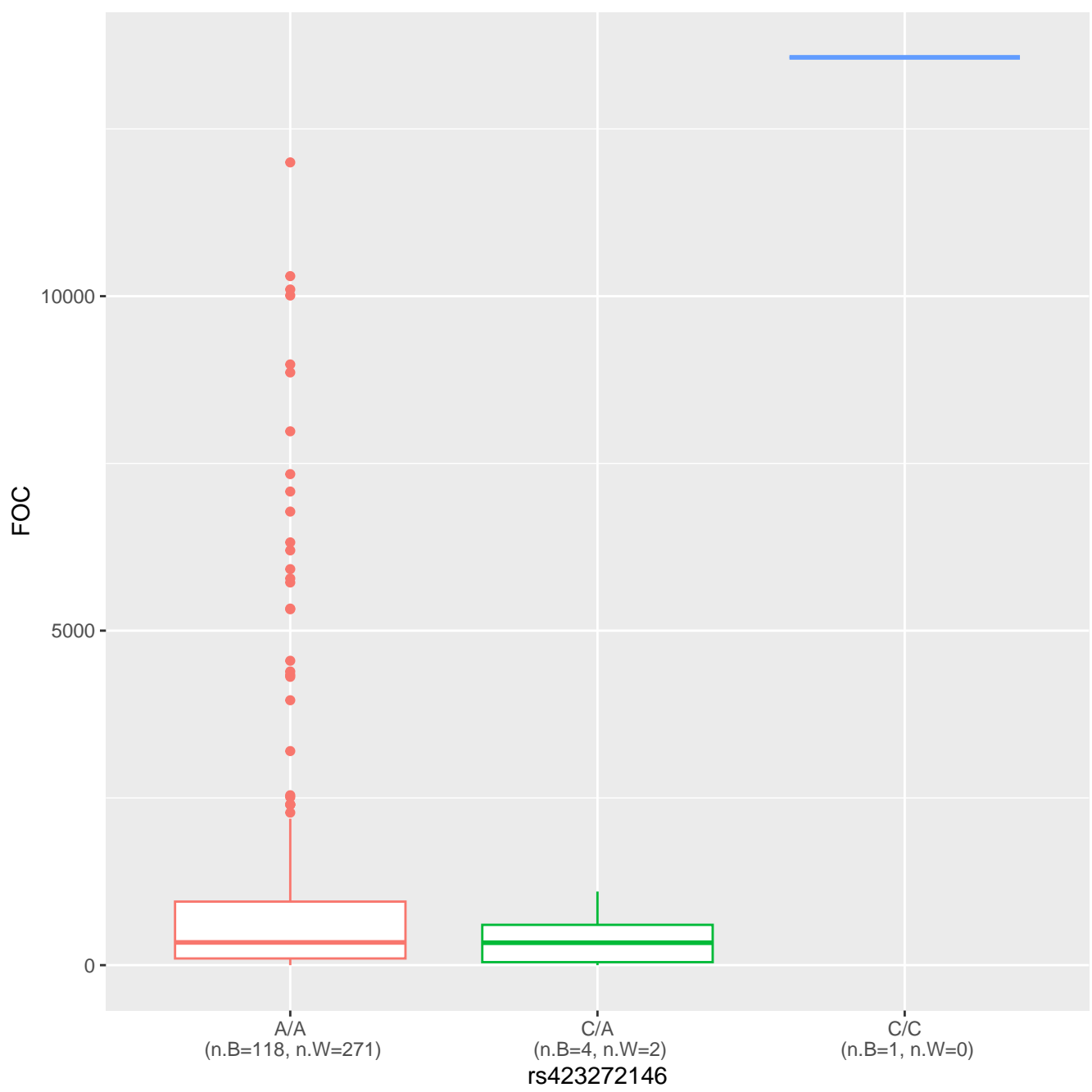

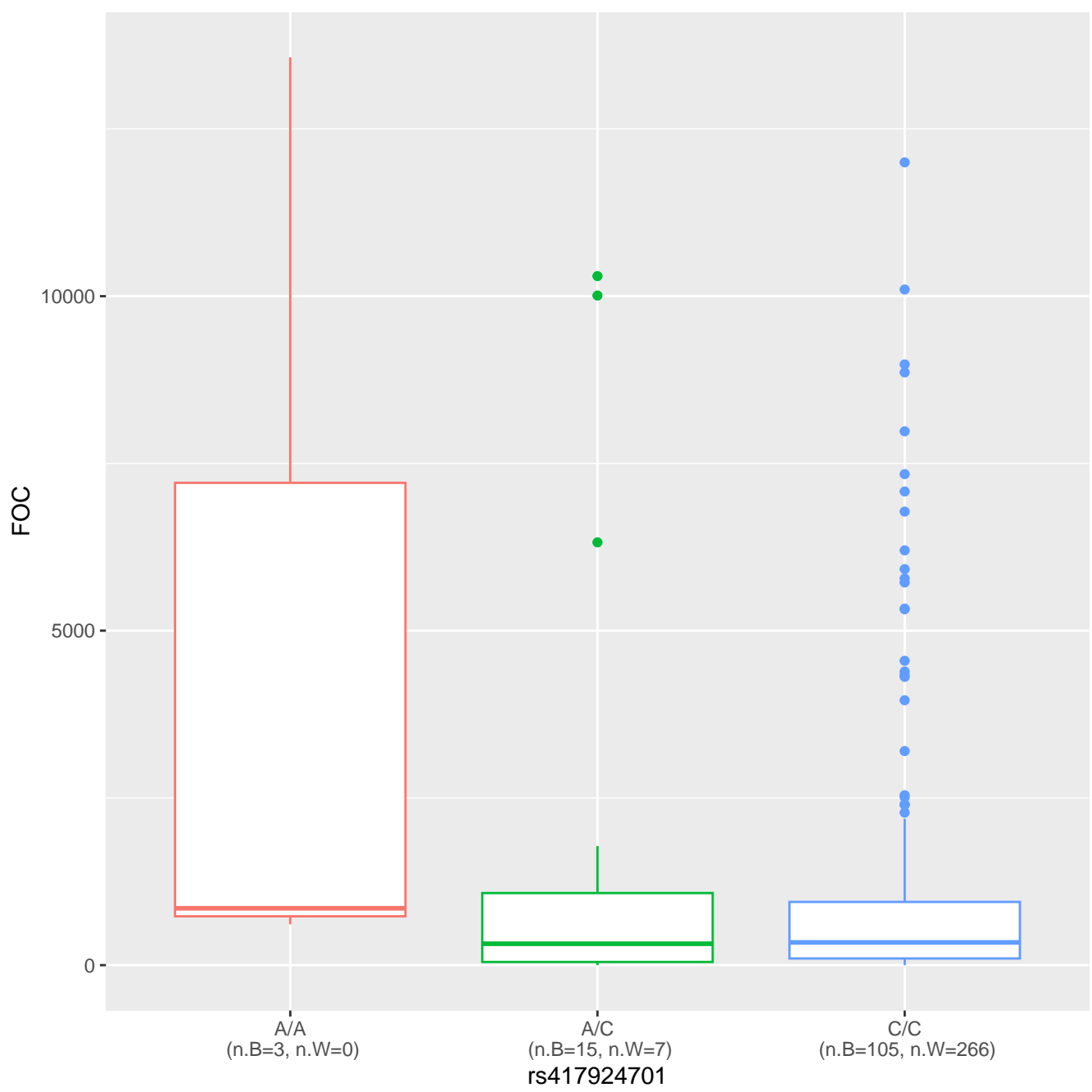

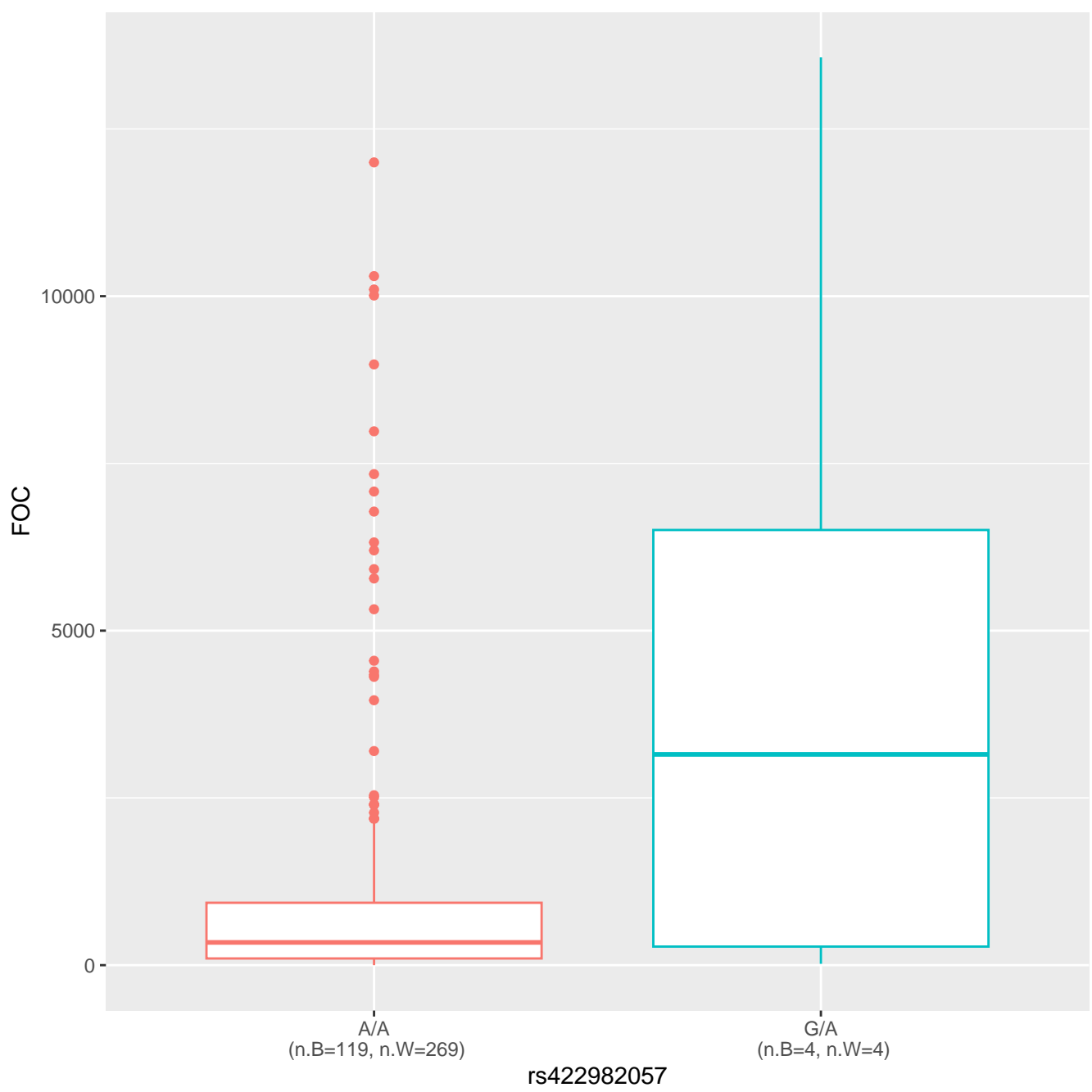

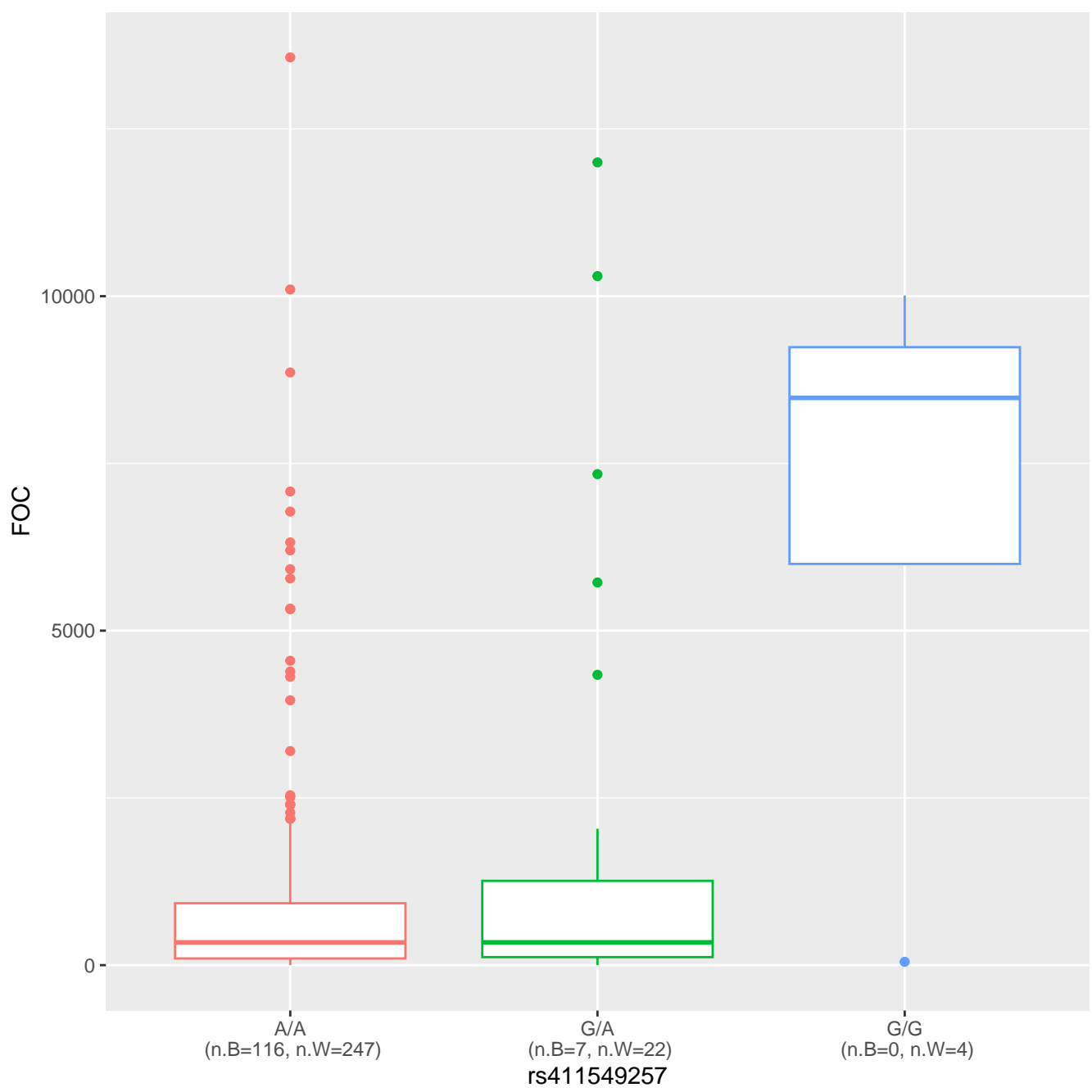

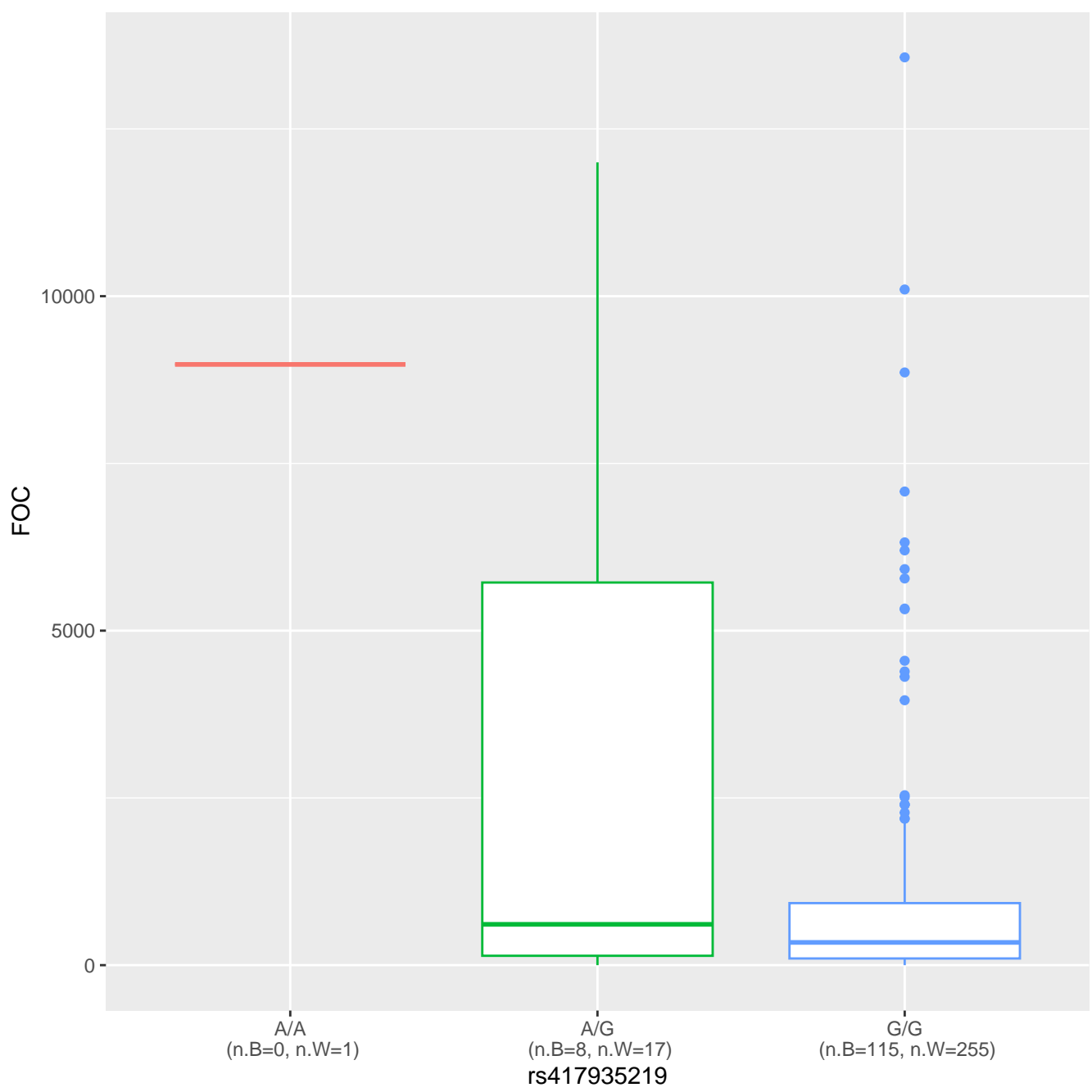

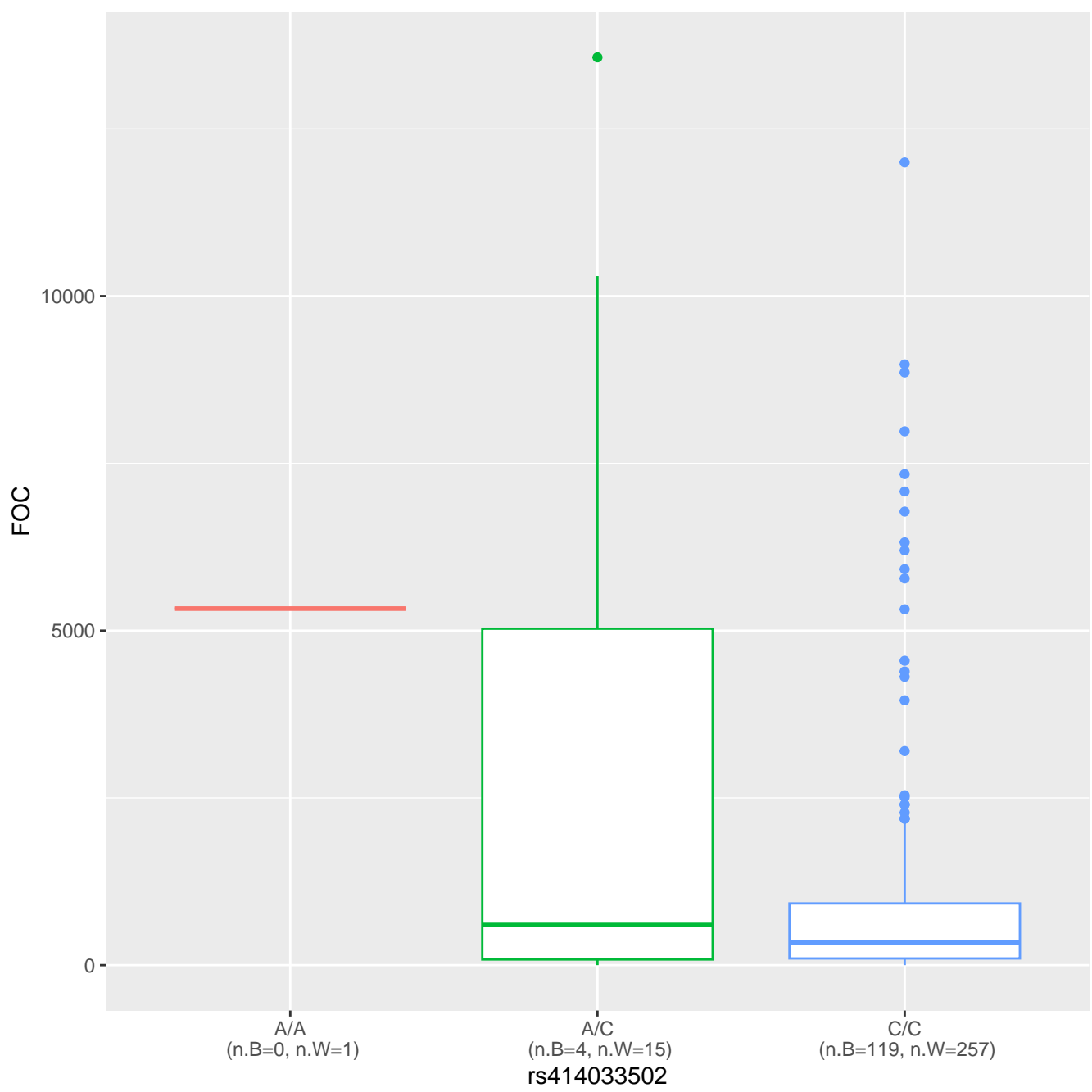

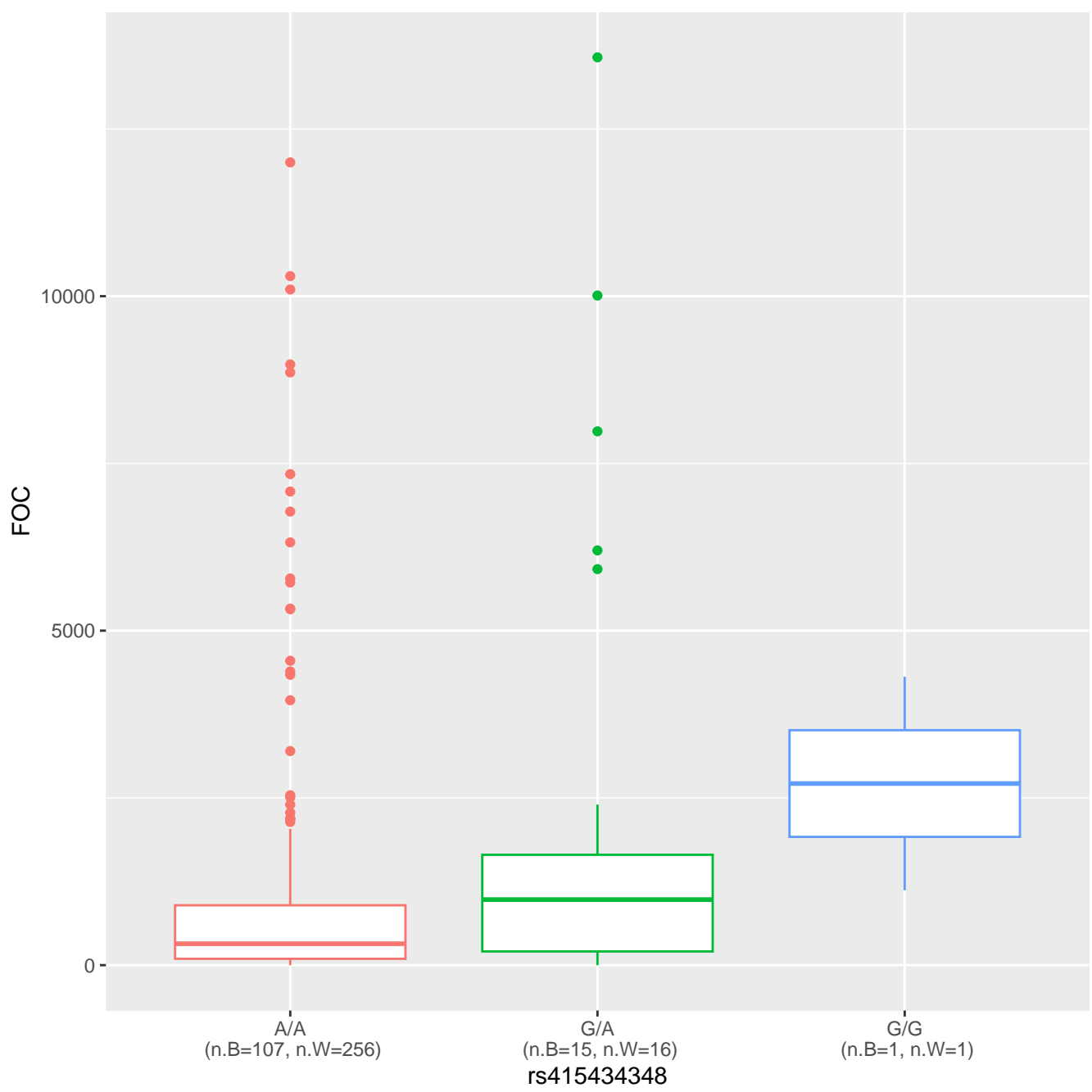

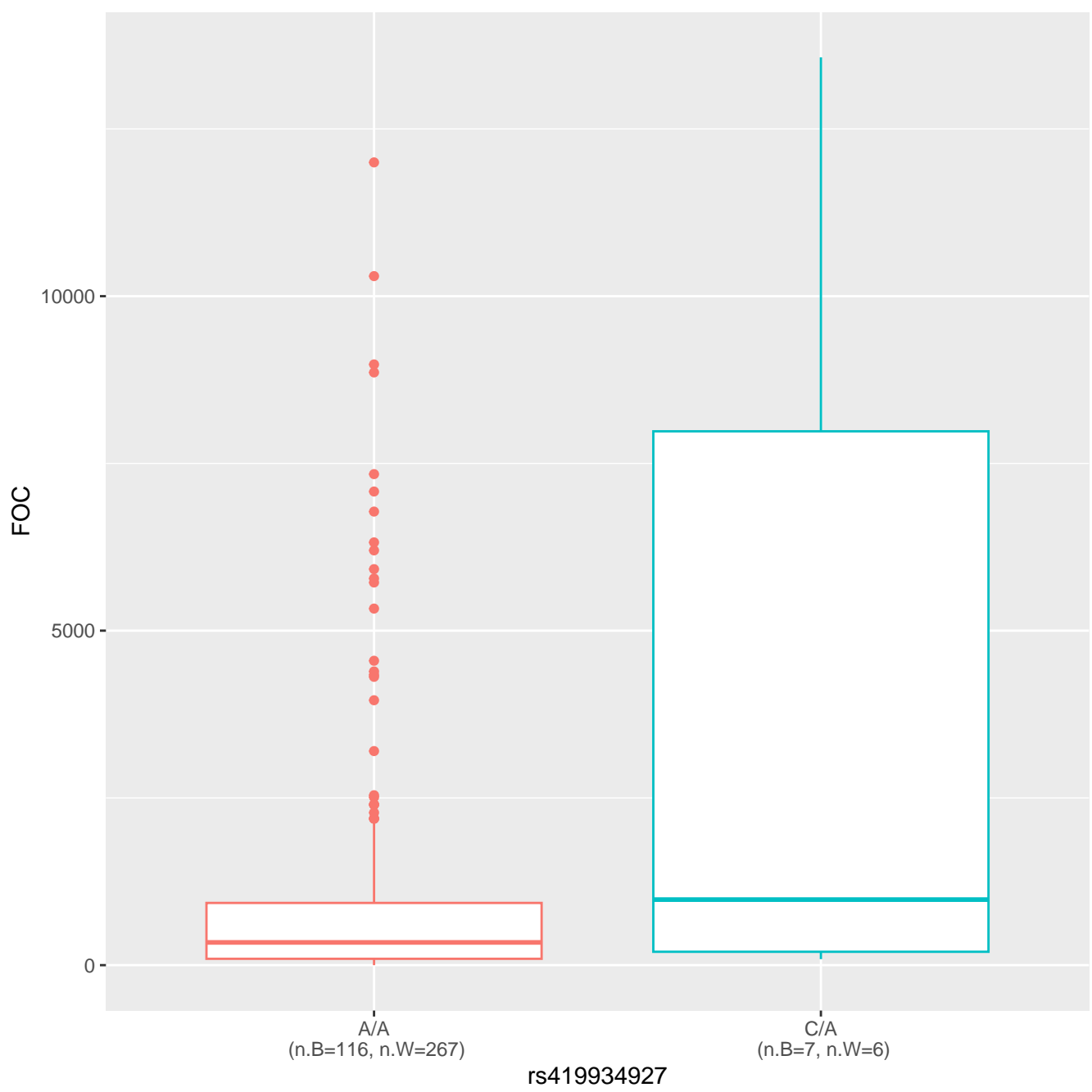

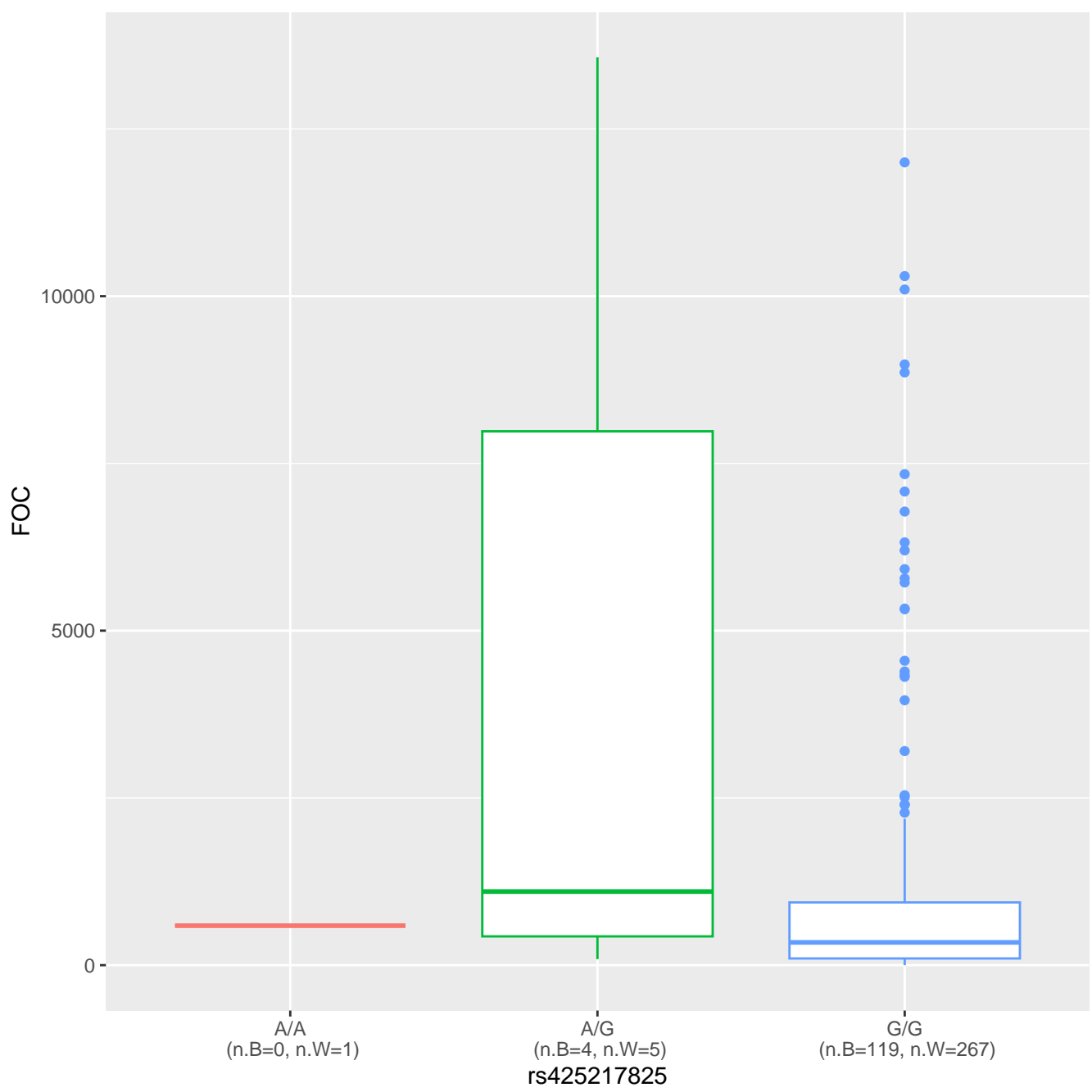

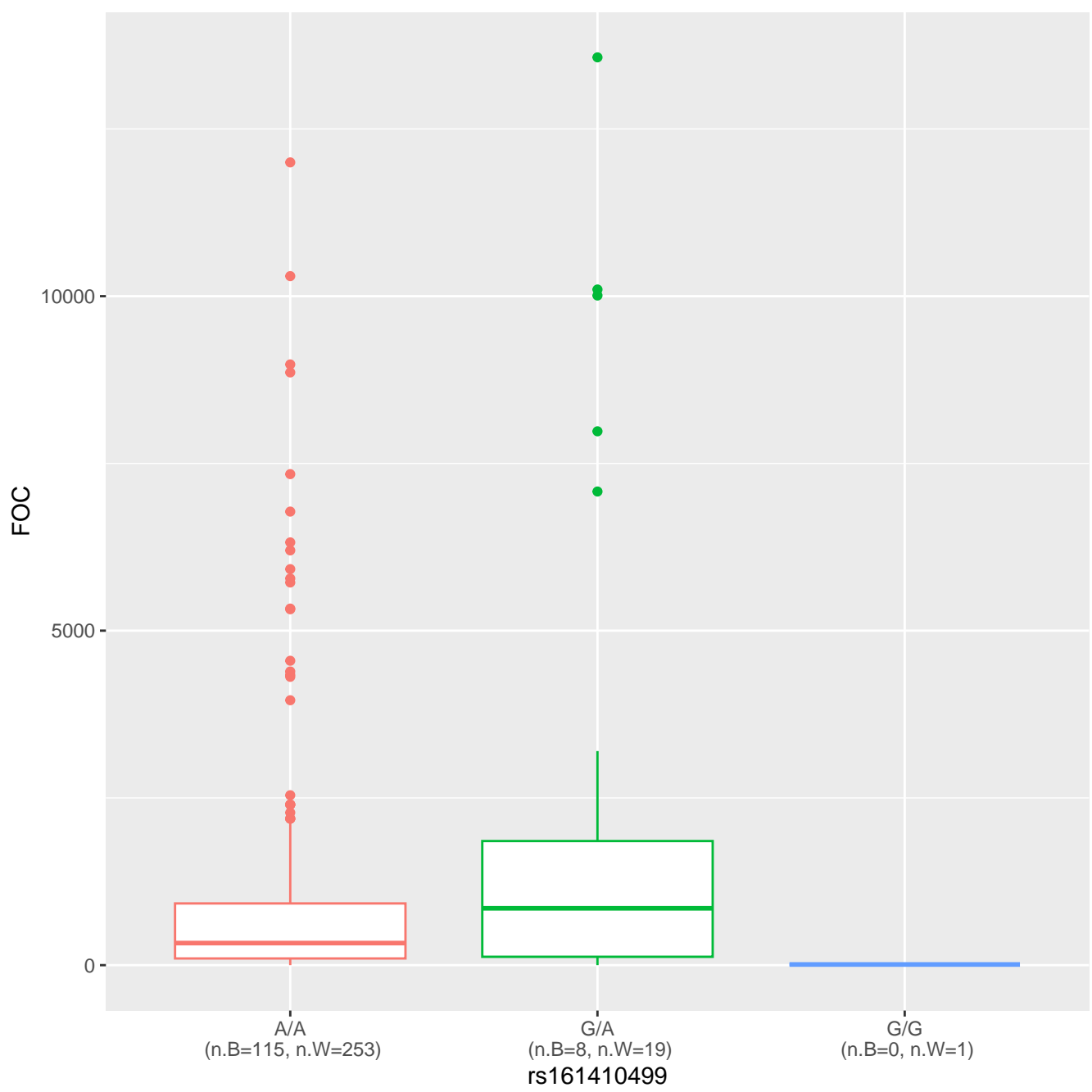

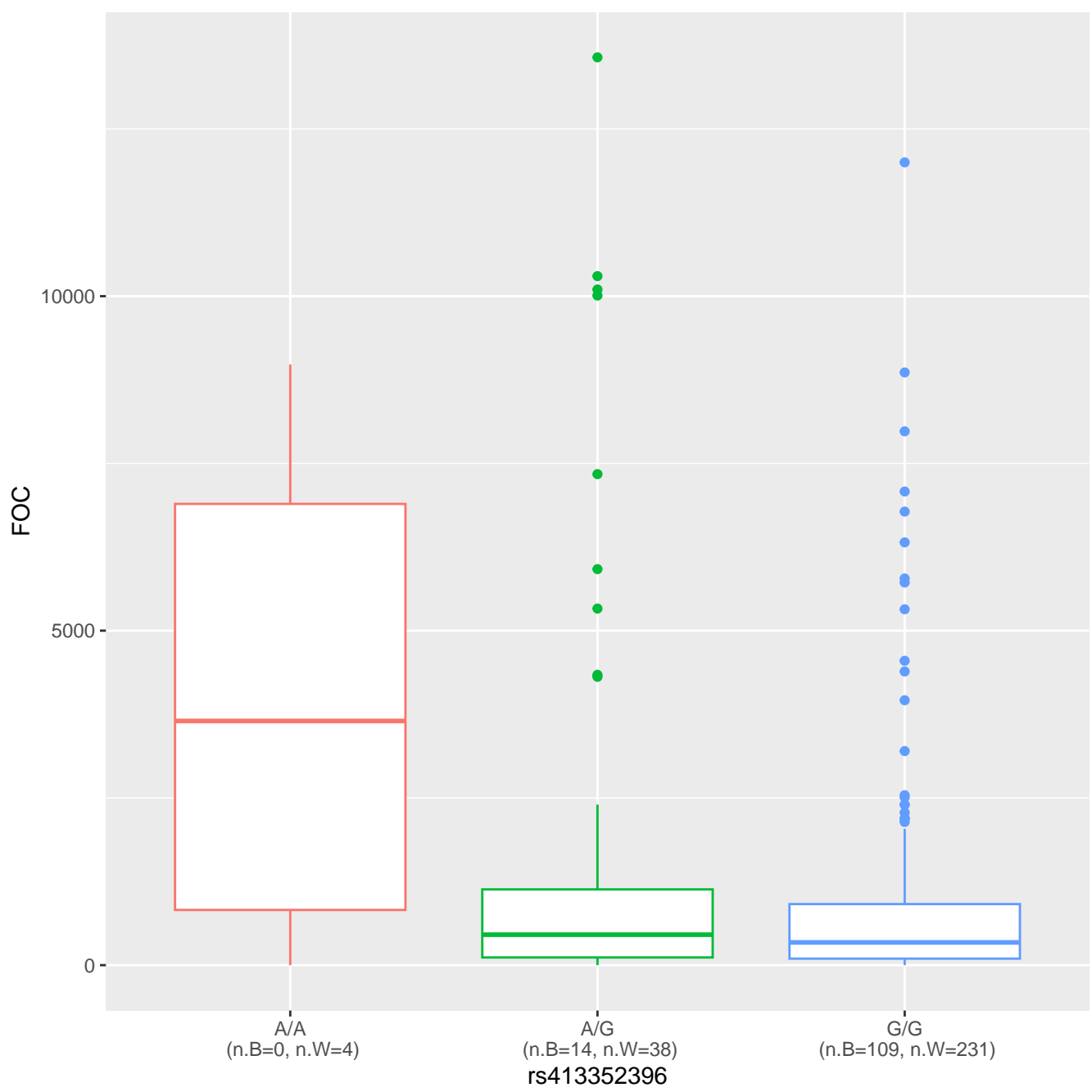

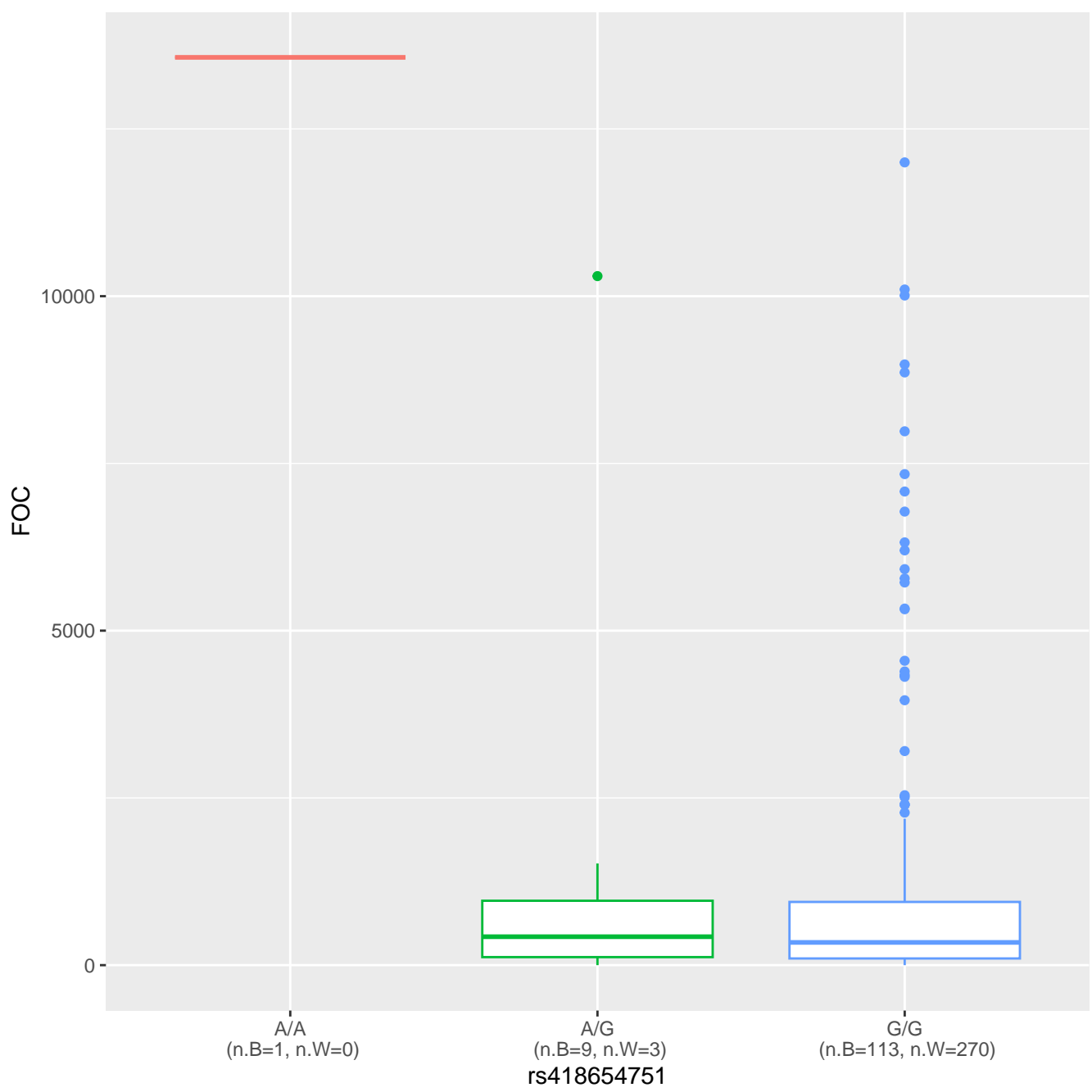

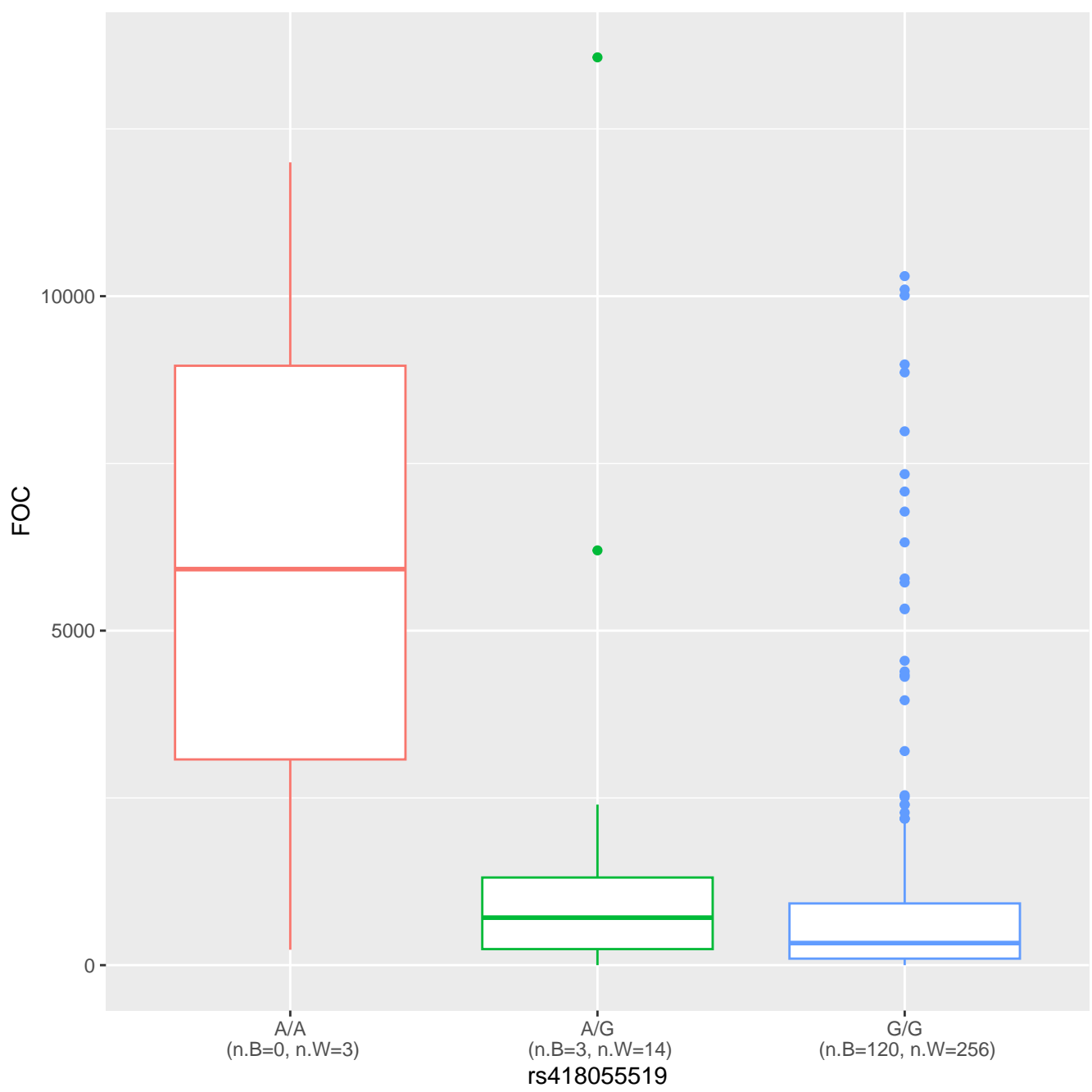

Supplement: Supplementary file 10 — Supplementary Material 10: Boxplots showing genotype distribution for the significant SNPs identified in this study. The marker ID can be seen at the bottom. The genotype in the x-axis and FOC values in the y-axis. n=number of Merino sheep. [file 12711_2025_1020_MOESM10_ESM.pdf]
